# The RelA/SpoT Homolog (RSH) Superfamily: Distribution and Functional Evolution of ppGpp Synthetases and Hydrolases across the Tree of Life

**DOI:** 10.1371/journal.pone.0023479

**Published:** 2011-08-09

**Authors:** Gemma C. Atkinson, Tanel Tenson, Vasili Hauryliuk

**Affiliations:** University of Tartu, Institute of Technology, Tartu, Estonia; East Carolina University, United States of America

## Abstract

RelA/SpoT Homologue (RSH) proteins, named for their sequence similarity to the RelA and SpoT enzymes of *Escherichia coli*, comprise a superfamily of enzymes that synthesize and/or hydrolyze the alarmone ppGpp, activator of the “stringent” response and regulator of cellular metabolism. The classical “long” RSHs Rel, RelA and SpoT with the ppGpp hydrolase, synthetase, TGS and ACT domain architecture have been found across diverse bacteria and plant chloroplasts, while dedicated single domain ppGpp-synthesizing and -hydrolyzing RSHs have also been discovered in disparate bacteria and animals respectively. However, there is considerable confusion in terms of nomenclature and no comprehensive phylogenetic and sequence analyses have previously been carried out to classify RSHs on a genomic scale. We have performed high-throughput sensitive sequence searching of over 1000 genomes from across the tree of life, in combination with phylogenetic analyses to consolidate previous *ad hoc* identification of diverse RSHs in different organisms and provide a much-needed unifying terminology for the field. We classify RSHs into 30 subgroups comprising three groups: long RSHs, small alarmone synthetases (SASs), and small alarmone hydrolases (SAHs). Members of nineteen previously unidentified RSH subgroups can now be studied experimentally, including previously unknown RSHs in archaea, expanding the “stringent response” to this domain of life. We have analyzed possible combinations of RSH proteins and their domains in bacterial genomes and compared RSH content with available RSH knock-out data for various organisms to determine the rules of combining RSHs. Through comparative sequence analysis of long and small RSHs, we find exposed sites limited in conservation to the long RSHs that we propose are involved in transmitting regulatory signals. Such signals may be transmitted via NTD to CTD intra-molecular interactions, or inter-molecular interactions either among individual RSH molecules or among long RSHs and other binding partners such as the ribosome.

## Introduction

Bacteria use several modified nucleotides as intracellular messengers, such as cAMP, c-di-GMP, c-di-AMP, cGMP, and ppGpp, with the latter being the first to be identified and one of the best studied [1], [2]. RelA/SpoT Homologue (RSH) proteins [3], such as the RelA and SpoT proteins of *Escherichia coli*, regulate the concentration of the alarmone ppGpp (guanosine 5′-diphosphate, 3′-diphosphate) in response to various environmental cues such as temperature change [4], [5], transition to the stationary phase [6], carbon [7], iron [8], fatty acid [9], phosphate [10] and amino acid limitation [11]. The cellular stress response mediated by increased ppGpp levels is referred to as the “stringent response” (reviewed in [12]).

The first type of stringent response to be investigated was the RelA-mediated response to amino acid limitation in *E. coli* [13], [14]. Under these conditions, accumulation of deacylated tRNA bound in the ribosomal A-site is sensed by RelA. This protein uses ATP and GDP (or GTP) to synthesise the alarmone nucleotide ppGpp (or pppGpp) in a synthetase (herein referred to as SYNTH) domain [11]. The pppGpp nucleotide is rapidly degraded *in vivo* to ppGpp by the enzyme pppGpp phosphohydrolase (GPP) [15]. Translational GTPases EF-G, EF-Tu and IF2 also catalyze pppGpp degredation *in vitro* [16], [17], however, the physiological significance of this is not clear. *E. coli* has a second RSH protein, SpoT, which is bifunctional, with weak ppGpp synthetic activity in its SYNTH domain [18] and strong ppGpp degrading activity mediated by a hydrolysis (HD) domain. The HD domain is also present but inactive in RelA [19], [20]. RelA and SpoT are found in γ- and β- proteobacteria and are thought to have evolved via gene duplication of an ancestral Rel protein found in many groups of bacteria [3]. Like SpoT, Rel is bifunctional with active SYNTH and HD domains [21], [22], [23], [24], [25]. Unfortunately, there is considerable confusion in the nomenclature in the current literature, with Rel, RelA, Rsh, RelA and SpoT often being used interchangeably for orthologous proteins from different organisms (for example [25], [26]). Here, we use the nomenclature Rel for the ancestral bifunctional RSH, and RelA and SpoT for the two homologs derived from Rel gene duplication in proteobacteria, as per [3], and refer to these three SYNTH and HD-containing proteins as “long RSHs”. In addition to the SYNTH and HD domains, long RSHs usually also carry TGS and ACT domains in their carboxy-terminal (CTD) region. The precise function of these domains is unclear, but they are involved in mediating inter- and intra molecular interactions and regulating catalytic activity [12].

Along with long RSHs, shorter and specialized RSHs (“short RSHs”) that contain either the SYNTH or the HD domain have been identified. SYNTH domain-only proteins have been found in firmicute bacteria (RelP and RelQ also known as Yjbm and Ywac, respectively) [27], [28] and *Vibrio cholera* (RelV) [29]. HD domain-only forms have so far not been reported in bacteria, but have been identified in metazoa (Mesh1) [30]. Mesh1 is the only RSH described in eukaryotes, with the exception of plants, which encode multiple long RSHs that function in the chloroplast and act in response to stress [31].

Just as there are many different RSH proteins, there are many targets of ppGpp in the cell. Thus, modulation of the intracellular ppGpp concentration acts as a hub, regulating transcription [32], translation [33], [34], acid stress response [35], replication [36] and in general serving as one of the main homeostatic instruments adjusting bacterial cell growth rate [37] (reviewed in [38]). These pleiotropic effects of ppGpp tightly link the stringent response with virulence of many bacterial pathogens, making it of considerable medical interest (reviewed in [39]). However, despite the complexity and importance of the RSH-mediated stringent response, there is no comprehensive knowledge of the distribution of RSHs, the core enzymes of ppGpp metabolism. The only previous dedicated evolutionary analysis of Rel, RelA and SpoT overlooked more divergent forms, including the small RSHs of firmicutes and *Vibrio* [3]. This analysis was performed almost a decade ago, when few genomes were available and intensive phylogenetic analyses were not as feasible. Therefore, we have conducted a timely up-to-date large-scale, comprehensive sequence and phylogenetic analysis of this superfamily, taking advantage of the available sequence data from many bacteria, eukaryotes and archaea, and using Hidden Markov Models (HMMs) for sensitive sequence searching.

We have identified and classified RSHs and their domain architectures from available genomes across the tree of life to retrace the evolution of RSH form and function using the structural and functional information available for these proteins. The crystal structure of the Rel SYNTH and HD domains from *Streptococcus equisimilis* (“Rel_Seq_”) has been determined [40], while mutational analyses have identified sites important for synthetase and hydrolase activities [40], oligomerization [41], [42], interactions with the ribosome [41], interdomain cross-talk [43] and nucleotide binding [24], [44]. In the absence of high-resolution structural data of RSH:ribosome complexes as well as full length RSH proteins from different subgroups, our understanding of the architecture of these functionally important sites is quite rudimentary. Thus, *in silico* analyses are valuable for predicting direct links between sequence and function, generating hypotheses that can be tested biochemically. By comparative analysis of patterns of sequence variation among RSHs, we have identified sites that we propose are involved in transmitting signals from the CTD region or from other interacting molecules. We suggest the Rel, RelA and SpoT system is an interesting case study for examining the fate of gene duplicates, as they show domain specific features of subfunctionalization and specialization.

## Methods

Sequence dataset assembly was an iterative process, beginning with a first-pass survey of the RSH superfamily tree from sequences stored in the Pfam database, and followed by two iterations of Hidden Markov Model (HMM) searching against completed genomes. For the first-pass survey, the full alignment of the synthetase domain was downloaded from Pfam (RelA_SpoT, PF04607 [45]). This dataset was reduced by eye to remove duplicate and highly similar sequences from closely related organisms. Sequences were aligned using MAFFT version 6.626b [46]. In order to establish the general structure of the superfamily and identify initial subgroups, a preliminary tree RaxML version 7.0.4 [47] maximum likelihood (ML) tree was generated. To limit the presence of missing data in the alignments, sequence fragments of <100 amino acids were removed before all phylogenetic analyses. Such truncated sequences are rare (31 of 2077 sequences in the final RSH database). To select regions of the alignment suitable for phylogenetic analysis, the alignment was trimmed to columns containing <50% gaps using the Consensus Finder Python script [48] and curated by eye to ensure non-aligned regions were not included. RaxML was run on the Cipres server version 2.2 (http://www.phylo.org/sub_sections/portal/) with the PROTCATWAG model, with 100 bootstrap replicates. From this starting tree, 15 apparent paralogous subgroups (distinct clusters of sequences) were identified. Hidden Markov Models (HMMs) were created with HMMER 3.0b2 (http://hmmer.org/, [49]) for each subgroup with 4 or more taxa in order to find more members of each subgroup in the subsequent step.

The predicted proteomes from 1072 organisms with complete genomes were downloaded from NCBI (http://www.ncbi.nlm.nih.gov/Ftp/), the DOE Joint Genome Institute (JGI; http://genome.jgi-psf.org/), and the *Cyanidioschyzon merolae* Genome Project (CMGP; http://merolae.biol.s.u-tokyo.ac.jp/) (organism source listed in Table S1 and taxonomy in Table S2). All sequences were searched against the collection of RSH HMMs from the previous round, plus the original RelA_SpoT Pfam domain HMM. The results were stored and organized in a local MySQL database. An initial E value cut-off of E^−2^ for the RelA_SpoT domain was used as the gathering threshold, resulting in hits to 2196 sequences. As the most distant homologs could not be reliably aligned across their full length, only those sequences with E values less than E^−5^ were selected for phylogenetic analyses. Sequences were aligned and ML phylogenetic trees were generated as above. Visual inspection of the resulting tree identified seven additional subgroups. The HD (hydrolysis) domain from Pfam (PF01966) was also added at this stage in order to identify HD-containing proteins that may not necessarily be accompanied by a RelA_SpoT synthetic domain. An E value cut off of E^−2^ was also used for genome searches using the HD domain HMM. From the results of phylogenetic analysis as described above, we classified sequences into six HD-only subgroups, from which sequences were aligned and HMMs created. From the results of these searches, the HD and RelA_SpoT domain HMMs were remade and genomes rescanned with these and the 30 subgroup HMM models. In order to avoid confusion arising from the rather ambiguous name “RelA_SpoT” as used in Pfam for the ppGpp syntetase domain, the remade “RelA_SpoT” HMM is henceforth referred to as the SYNTH domain HMM.

After examining the results of the HMM scan, the results were filtered in order to remove spurious SYNTH and HD domain hits to distant relatives. These spurious hits include the RNA binding domain of DEAD-box helicases in the case of the HD domain, and ribosomal protein L17 and the NADP-binding domain of glutamyl tRNA reductase in the case of the SYNTH domain. The new E value filtering thresholds were set at E^−4^ and E^−5^ for SYNTH and HD domains, respectively. From the resulting datasets, sequences were aligned and trees created as above. As the SYNTH domain alone is particularly short (mean length 113 amino acids), additional NTD and CTD domains were used in this alignment to help resolve relationships in the long RSH part of the tree, with missing data coded for the SAS sequences in short poorly aligned regions upstream and downstream of the SYNTH domain. Final dataset dimensions were 670 amino acid positions from 1706 sequences for the SYNTH-containing data set and 168 amino acid positions from 1535 sequences for the HD-containing dataset. Phylogenetic analysis was also carried out separately on sequences that contain both the SYNTH and HD domains (1223 sequences, 699 positions). Inspection of the final trees allowed classification of all the sequences into 30 subgroups.

In order to examine branching patterns of subsets of the data more clearly, additional, separate phylogenetic analyses were conducted on the chloroplast RSHs (470 amino acid positions from 66 sequences) and the Mesh1/Mesh1-L subgroups (179 amino acid positions from 99 taxa). Phylogenetic analysis was carried out using RaxML as above, and with Bayesian inference, using MrBayes version 3.1.2 [50] on the Cipres server 2.2. MrBayes was run with 8 chains, with a gamma plus mixture model (which converged on the WAG substitution model with probability 1.0 in both cases) for two million generations. At the end of the run, the standard deviation of split frequencies was 0.003 for the chloroplast sequences and 0.04 for Mesh1/Mesh1-L sequences. A consensus tree was generated from 30000 trees, after discarding the first 5000 trees from each of the two runs as a burn in.

Consensus sequences from aligned sequences were generated using the Consensus Finder Python script to generate consensus alignments for analyzing differential conservation among subgroups [48]. Identical sequences were removed from the alignment before generating the consensuses, and a 70% percentage similarity threshold was used. DIVERGE 2.0 [51], [52], [53] was used to test for type I and type II functionally divergent sites between RelA and SpoT subgroups. Type I functional divergence refers to changes in the amino acid substitution rates of homologous sites among distinct clades of sequences. In type II functionally divergent sites, amino acid substitutions are completely fixed between duplicates and result in radical shifts in physiochemical properties. The full length RelA/SpoT/Rel dataset multiple sequence alignment was reduced down to a set of 97 sequences that could be used as input to DIVERGE, and a rooted NJ tree was generated by the software using the Poisson distance correction distance measure (tree shown in Figure S1). Monophyletic clusters of RelA and SpoT were selected and used to calculate the coefficient of functional divergence (theta) and the posterior probability (PP) that a site is functionally divergent for each column of the alignment (columns containing gaps are automatically excluded by the program). We used a cut-off of 0.90 posterior probability to indicate a strong probability of the site being functionally divergent

Secondary structure and regions of structural disorder were predicted with Psipred and Disopred respectively, using the Disopred server (http://bioinf.cs.ucl.ac.uk/disopred) [54]. The TargetP server was used for predicting subcellular location (http://www.cbs.dtu.dk/services/TargetP/) [55]. Structures were visualized with MacPyMol [56].

## Results

### Distribution of the RSH superfamily

**Figure 1:**
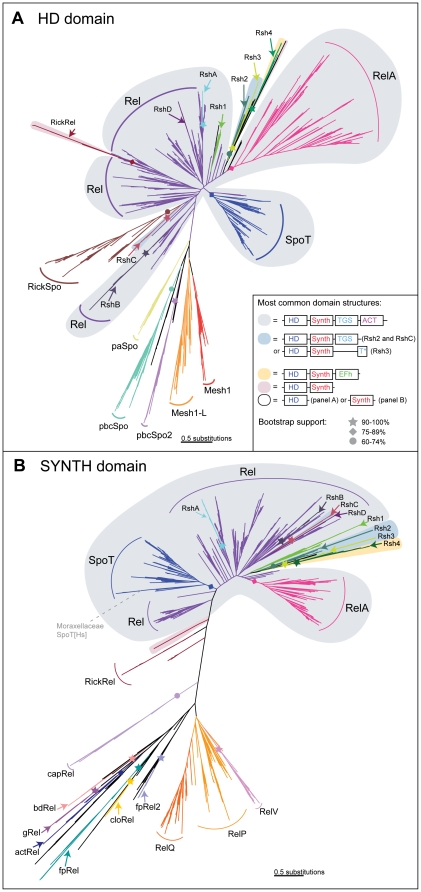
Maximum likelihood phylogenies of the ppGpp synthetase and hydrolase domains. Trees were generated from RaxML analyses of alignments of A) the ppGpp hydrolase (HD) domain-containing dataset (168 amino acid positions, 1535 sequences), and B) the ppGpp synthetase (SYNTH) domain-containing dataset (670 amino acid positions, 1706 sequences). In both trees, subgroups are labeled and shading behind the branches shows the most common domain structure observed for those groups, as per the legend in the inset box. Symbols on branches indicate bootstrap support, as per the inset box. In cases where the whole subgroup carries both the HD and SYNTH domain (Rel, SpoT, RelA, Rsh1-4, RshA-D), bootstrap support comes from the full length long RSH tree (supplementary file SI2). Branch length is proportional to the number of substitutions per site (see scale bar).

Iterative HMM searching of 1072 complete genome sequences and phylogenetic analyses enabled the identification of RSH sequences from genomes across the tree of life (Tables 1, S1 and S2). RSH proteins are defined by the presence of a ppGpp synthetase (SYNTH, corresponding to the “RelA_SpoT” domain of Pfam) domain and/or a ppGpp hydrolase (HD) domain. Maximum likelihood phylogenetic trees of SYNTH-carrying and HD-carrying data sets show the diversity of RSHs (Fig. 1A and B). Proteins were classified into subgroups based on clustering of sequences in phylogenetic analyses that tend to have single representatives in one genome, thus suggesting they may represent orthologous subfamilies. Most subgroups have moderate to strong statistical support for monophyly, i.e. common descent from a single ancestor (maximum likelihood bootstrap support (MLBP) of over 60% for 20 subgroups, over 75% for 14 subgroups and over 90% for 11 subgroups, as shown in Figures 1A and B). However, it is not possible to achieve strong statistical support for monophyly for all subgroups given the necessarily few sites used to build the small RSH-containing phylogenies, in combination with the considerable sequence divergence of many RSHs. It is likely that as more genomes are sequenced, the subgroup resolution is likely to improve.

**Table 1:** The 30 subgroups of RSHs, their taxonomic distributions and additional descriptions.

Type	Subgroup	Taxonomic distribution (major groups represented)	Description
Long RSHs	Rel	α/δ/ε-proteobacteria, Acidobacteria, Synergistetes, Aquificae, Bacteroidetes, Chlorobi, Spirochaetes, Actinobacteria, Cyanobacteria, Chloroflexi, Deinococcus/Thermus, Firmicutes, Tenericutes, Fusobacteria	Bifunctional hydrolases and synthetases. The original long RSH ortholog from which other long RSHs evolved by gene duplication or HGT
	SpoT	γ/β-proteobacteria	Largely bifunctional. Some monofunctional hydrolases
	RelA	γ/β-proteobacteria	Monofunctional synthetases
	RshA	Actinobacteria	
	RshB	Bacteroidetes	
	RshC	Bacilli	Some lack the ACT domain
	RshD	δ-proteobacteria	
	Rsh1	Archaeplastida, Haptophycaea	
	Rsh2	Archaeplastida, Haptophycaea	
	Rsh3	Archaeplastida, Haptophycaea	
	Rsh4	Archaeplastida, Haptophycaea	Carry an EF-hand calcium-binding domain
SAS	actRel	Actinobacteria	Some carry an N terminal domain of unknown function (“DUF429”)
	bdRel	β/δ- proteobacteria	
	cloRel	Firmicutes	
	fpRel	Firmicutes, α/β-proteobacteria	
	fpRel2	Firmicutes, β-proteobacteria	
	RelV	γ-proteobacteria	
	gRel	γ-proteobacteria	
	capRel	α/β/γ/δ -proteobacteria, cyanobacteria, actinobacteria	
	RickRel	α-proteobacteria	
	RelP	Firmicutes	aka Yjbm
	RelQ	Firmicutes	aka Ywac
	divRel	Archaea (*Methanosarcina acetivorans*), Eukaryotes (*Dictyostelium*, Ascomycota), Spirochetes	Miscellaneous unplaced sequences containing only the synthetase domain
SAH	paSpo	α/β/γ/δ-proteobacteria, Acidobacteria	
	pbcSpo	Archaea (*Natronomonas pharaonis*, uncultured methanogen)α/δ/γ-proteobacteria, Bacteroidetes, Cyanobacteria, Firmicutes, Actinobacteria	
	pbcSpo2	β/δ/γ-proteobacteria, Bacteroidetes, Cyanobacteria, Chloroflexi	
	Mesh1	Eukaryotes, α/β/δ-proteobacteria	Widespread in opisthokont eukaryotes
	Mesh1-L	Archaea (*Methanococcoides burtonii*), α/γ/δ-proteobacteria, Cyanobacteria, Actinobacteria, Bacteroidetes, Firmicutes, Chloroflexi	
	RickSpo	α-proteobacteria, Bacteroidetes	Multiple duplicates in Rickettsiales and *Cand. Amoebophilus asiaticus*
	divSpo	Archaea (*Methanosarcina barkeri*), Eukaryotes (*Aureococcus anophagefferens, Emiliania huxleyi),* γ/δ-proteobacteria, Cyanobacteria, Firmicutes,	Miscellaneous unplaced sequences containing only the hydrolase domain

RSH subgroups can be grouped into three classes: Small Alarmone Synthetases (SAS, [27]) that contain only the SYNTH domain, Small Alarmone Hydrolases (SAHs) containing only the HD domain, and longer proteins that carry the SYNTH and HD domains, with or without additional CTD domains (long RSHs). Within these classes are 11 subgroups of long RSHs (Rel, SpoT, RelA, RshA, RshB, RshC, RshD and Rsh1-4), 12 groups of SASs (actRel, bdRel, cloRel, fpRel, fpRel2, RelV, gRel, capRel, rickRel, RelP, RelQ and divRel), and 7 groups of SAHs (paSpo, pbcSpo, pbcSpo2, Mesh1, Mesh1L, rickSpo, divSpo) (Tables 1, S1 and S2). The long RSHs RshB, RshC, and RshD are groups of proteins nested within the Rel subgroup that occur as more divergent second copies in their encoding organisms, suggesting they originated by gene transfer or duplication. They are named to continue the nomenclature previously used for RshA [25], also a Rel gene duplicate. Small RSHs are named according to previous descriptions, or if they are undescribed, a prefix is given that refers to the main distribution of the group, followed by “Rel” if the protein is a synthetase, and “Spo” if the protein is a hydrolase. From herein, when discussing subgroups in the text, we use square brackets to indicate the presence of the HD [H] or SYNTH [S] domain in the protein. For example fpRel[S] is an SAS found in firmicutes and proteobacteria. Although RelA has not been found capable of ppGpp hydrolysis, it still carries an inactive HD domain and so is referred to as RelA[hS]. The sequence divergence of the inactive RelA[hS] hydrolase domain can be seen in the relative branch lengths between the RelA clades of the HD and SYNTH trees (Fig. 1A and B). Miscellaneous divergent SASs and SAHs that can not be assigned to any other particular subgroup are referred to as divRel[S] and divSpo[H], respectively.

The average currently sequenced bacterial genome encodes two RSHs in its genome, even if the β- and γ-proteobacteria that encode RelA[hS] and SpoT[HS] are excluded. By far the most widespread RSH is Rel[HS]. Although there has been some confusion in the literature, using the name Rel as a synonym for RSH, we follow the convention of Mittenhuber *et al.* [3], where Rel is the name for the ubiquitous classical bifunctional RSH protein that was the precursor of RelA and SpoT. By use of the name Rel, we also support the terminology used for the two most well studied bifunctional RSHs Rel_Seq_ and Rel_Mtb_, which are both Rel[HS] proteins. We suggest that this nomenclature, where the protein name is ended with a subscript abbreviation of the species name is a useful convention when discussing RSHs from specific species.

Due to its wide taxonomic distribution and paraphyly with respect to other clades of long RSHs (SpoT[HS], RelA[hS], RshA[HS], RshB[HS], RshC[HS] and RshD[HS], Fig. 1A, 1B and S2), Rel[HS] appears to be the ancestral long RSH, which was the antecedent of all other bacterial long RSHs. Rel[HS] is found in all major groups of bacteria, with the exception of chalmydiales, verrucomicrobia and plantomycetes (the PVC superphylum [57], [58]) (Tables 1 and 2). Apart from the PVC bacteria, 35 bacterial species do not encode any RSH. These are mainly intracellular endosymbionts and pathogens in the genera *Bifidobacterium, Anaplasma, Ehlichia, Neorickettsia, Rickettsia, Wolbachia, Buchnera, Wigglesworthia* and *Baumania*, plus “Candidatus” bacteria *Blochmannia, Sulcia, Hodgkinia and Liberibacter*. Of free-living organisms, RSHs could not be found in seven of thirteen species of *Mycoplasma*, three of thirteen species of Spirochetes, and *Thermoanaerobacter* X514 of Clostridiales. One of the spirochete strains lacking an RSH, *Leptospira biflexa* strain “serovar Patoc 1 Paris” has a genomic hit to Rel[HS], but due to a CAA to TAA substitution, resulting in a premature stop codon, it is annotated as a pseudogene.

**Table 2:** Typical combinations of RSHs in bacteria.

Taxonomy		Typical combinations	N^o^ taxa in group	N^o^ RSHs	SpoT	RelA	Rel	RshA	RshB	RshC	RshD	*actRel*	*bdRel*	*cloRel*	*fpRel*	*fpRel2*	*RelV*	*gRel*	*capRel*	*rickRel*	*RelP*	*RelQ*	*divRel*	paSpo	pbcSpo	pbcSpo2	Mesh1	Mesh1-L	rickSpo	divSpo
					Long RSH							SAS												SAH						
Acidobacteria	Acidobacteria	Rel[HS]	1	1			x																							
Acidobacteria	*Cand. Koribacter*	Rel[HS],divRel[HS],Mesh1-L[H],paSpo[H]	1	4			x																x	x				x		
Acidobacteria	Solibacteres	Rel[HS]	1	1			x																							
Actinobacteria	Actinobacteria	Rel[HS] with additional SAS and/or SAH	79	1–4			x	x				x							x			x	x		x			x		
Aquificae	Aquificae	Rel[HS]	5	1			x																							
Bacteroidetes	Bacteroidia	Rel[HS], RshB[HS]	8	1–3			x		x														x							
Bacteroidetes	Cytophagia	Rel[HS],pbcSpo2[H]; Rel[HS],paSpo[H]	2	2			x																	x		x				
Bacteroidetes	Flavobacteria	Rel[HS],pbcSpo2[H]; Rel[HS],paSpo[H]; 0	10	0–2			x																	x		x				
Bacteroidetes	*Cand. Amoebophilus*	Rel[HS] + rickSpo[H] expansion	1	15			x																						x	
Bacteroidetes	Sphingobacteria	Rel[HS] + SAH expansion	4	1–4			x																	x	x	x		x		
Chlorobi	Chlorobia	Rel[HS]; Rel[HS],RelQ[S]; Rel[HS],Mesh1-L[H]	11	1–2			x															x						x		
Chloroflexi	Chloroflexi	Rel[HS]; Rel[HS],pbcSpo2[H]; Rel[HS],Mesh1-L[H]	6	2			x																			x		x		
Chloroflexi	Dehalococcoidetes	Rel[HS]	3	1			x																							
Chloroflexi	Thermomicrobia	Rel[HS]	1	1			x																							
Cyanobacteria	Gloeobacteria	Rel[HS],Mesh1-L[H]	1	2			x																					x		
Cyanobacteria	*Chroococcales*	Rel[HS] often + SAH, usually Mesh1-L[H]	19	1–4			x												x				x			x		x		x
Cyanobacteria	*Acaryochloris*	Rel[HS],pbcSpo[H]	1	2			x																		x					
Cyanobacteria	*Nostocales*	Rel[HS],Mesh1-L[H]; Rel[HS],Mesh1-L[H],pbcSpo2[H]	3	2–3			x																			x		x		
Cyanobacteria	*Oscillatoriales*	Rel[HS],Mesh1-L[H]	1	2			x																					x		
Cyanobacteria	*Prochlorales*	Rel[HS]	12	1			x																							
Deinococcus-Thermus	Deinococci	Rel; Rel[HS],divRel[S]	5	1–3			x																x							
Dictyoglomi	Dictyoglomia	Rel[HS]	2	1			x																							
Elusimicrobia		Rel[HS] or 0	2	1			x																							
Fibrobacteres	Fibrobacteres	Rel[HS],divRel[HS],divRel[HS],Mesh1-L[H]	1	4			x															x						x		
Firmicutes	Bacilli	Rel[HS],RelP[H],RelQ[H]	134	1–5			x			x					x	x					x	x			x			x		x
Firmicutes	Clostridia	Rel[HS] often + SAS, usually RelP[H],RelQ[H]	51	0–5			x							x	x						x	x	x		x					x
Fusobacteria	Fusobacteria	Rel; Rel[HS], fpRel[S]	2	1–2			x								x															
Gemmatimonadetes	Gemmatimonadetes	Rel[HS], Mesh1-L[H]	1	2			x																					x		
Nitrospirae	Nitrospira	Rel[HS]	1	1			x																							
Proteobacteria	α-proteobacteria	Rel[HS]; Rel[HS], Mesh1[H]; Rel[HS], capRel[S]	117	0–44			x								x			x	x	x				x	x	x	x	x	x	
Proteobacteria	β-proteobacteria	RelA[hS],SpoT[HS] often + SAS and/or SAH	73	1–4	x	x	x						x		x	x		x	x					x	x		x	x		x
Proteobacteria	δ-proteobacteria	Rel[HS] often + SAS and/or SAH	32	1–5			x				x		x		x				x				x	x	x	x	x	x		
Proteobacteria	ε-proteobacteria	Rel[HS]	25	1–2			x									x														
Proteobacteria	γ-proteobacteria	RelA[hS], SpoT[HS]	237	0–5	x	x							x		x	x	x	x	x					x	x	x		x		x
Proteobacteria	*Magnetococcus*	Rel[HS],rshD[HS],divRel[HS],pbcSpo[H]	1	4			x																x		x					
Spirochaetes	Spirochaetes	Rel; divRel[HS],divSpo[H] (T. denticola); 0	18	0–2			x																x							x
Tenericutes	Mollicutes	Rel; 0 (Mycoplasma)	24	0–1			x																							
Thermotogae	Thermotogae	Rel	10	1			x																							
Verrucomicrobia		zero	3	0																										
Chlamydiae	Chlamydiae	zero	15	0																										
Planctomycetes	Planctomycetacia	zero	1	0																										

Combinations are separated with semi-colons. “N^o^ taxa in group” indicates the number of taxa that have been reduced down the taxonomical groups in the following two columns. “N^o^ RSHs” shows the number or range of RSHs present in each genome of this taxonomical grouping. Presence of an RSH subgroup is indicated with “X”. Long RSHs are shown in bold, SASs in italic and SAHs in regular text.

### Phylogenetic and sequence analysis of long RSHs in bacteria

To better resolve relationships in the long RSH part of the tree using more homologous amino acid positions, maximum likelihood analysis was carried out on long RSHs (carrying at least the SYNTH and HD domains) alone. In this tree, branch support is 83% MLBP for RelA[hS] monophyly and 83% MLBP for SpoT[HS] monophyly (the full tree is shown in Figure S2, with branch support shown on Fig. 1A and B). The actinobacteria-specific RshA[HS] clade previously reported in *Streptomyces* [25] has 96% MLBP support for monophyly and groups with other actinobacteria Rel[HS] with 92% MLBP, suggesting it arose from a duplication of Rel in the actinobacteria lineage (Fig. S2). In addition to the RshA[HS] lineage-specific duplication of Rel[HS], we have identified three more subgroups within the Rel[HS] part of the tree (RshB[HS], RshC[HS] and RshD[HS], Fig. 1A, 1B, S2, Table 1). RshB[HS] is found in the genera *Bacteroides*, *Porphyromonas* and *Parabacteroides* of Bacteroidetes, and its nesting within other Bacteroidetes Rel[HS] sequences is well supported (98% MLBP), suggesting it arose from gene duplication of Rel[HS] within this class. Its long branch length in the HD domain tree (Fig. 1A) compared to the SYNTH domain tree (Fig. 1B) indicates considerable sequence divergence of RshB[HS] in the HD domain, similar to that for RelA. RshC[HS] is limited to a fully supported monophyletic group of six strains of *Bacillus*. Its origin is ambiguous, although it has reasonable support (76%) for being a sister group of an RshD[HS] protein of *Pelobacter carbinolocus.* RshD[HS]s are second copies of Rel[HS] that are found in four δ-proteobacteria (*Pelobacter carbinolocus, Desulfotalea psychrophilia, Syntrophobacter fumaroxidans and Magnetococcus MC-1*). Of these, only *Desulfotalea psychrophilia* and *Syntrophobacter fumaroxidans* form a significantly supported monophyletic group (100% MLBP). However, these sequences are classified as a subgroup by virtue of all being second copy RSHs from the same class of bacteria. As RshC[HS] and RshD[HS] have no support for their positions in the tree, they may be divergent paralogs, or could be xenologues, second copies of Rel[HS] that have originated via horizontal gene transfer (HGT).

**Figure 2:**
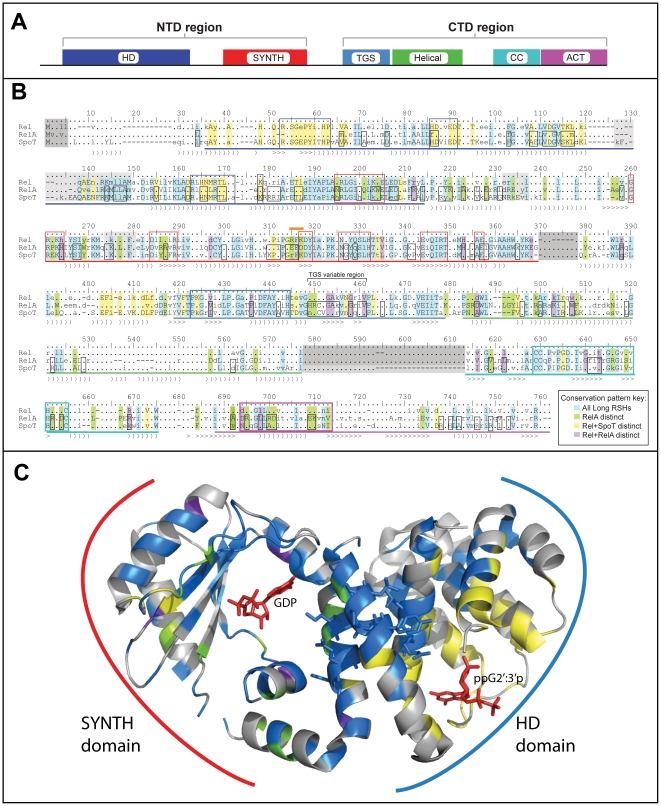
Consensus alignment of long RSHs, with Rel_Seq_ NTD structure colored according to conservation patterns in the alignment. A) Domain structure of the long RSHs, with domain lengths to scale with *S. equisimilis* Rel[HS]. B) Alignment of long RSH sequences at the 70% level. Secondary structure is shown below the alignment, with “)” characters indicating helices and “>” characters indicating sheets. Secondary structure is obtained from the structure of Rel [40] until position 362, after which second Psipred was used to predict the secondary structure. Disordered regions in the structure are underlaid with a pale grey box, and disordered regions predicted with Disopred are a darker grey. Highlighting of residue columns indicates conservation patterns (also see inset box). Blue highlighting indicates sites that are conserved across all long RSHs. Green highlighting shows those sites that are distinctive in RelA[hS] (strongly differentially conserved or conserved only in RelA[hS]). Yellow highlighted sites are well conserved in Rel[HS]+SpoT[HS] but less so RelA[hS], while purple highlighted sites are well conserved in Rel[HS]+RelA[hS] and less so in SpoT[HS]. Lines beneath the alignment indicate domains with the following colours: dark blue – HD, red – SYNTH, light blue – TGS, green – helical, turquoise – CC, magenta – ACT. Blue and red boxes show sites of the HD and SYNTH nucleotide binding pockets, respectively. Colored boxes in the TGS and ACT domain surround the usually most conserved blocks of these domains as per sequence logos in the Pfam database. The turquoise box in the CC domain indicates the most conserved block of this domain, which also contains the conserved cysteines of [59]. The orange bar above the alignment shows the location of the differentially conserved motif of [44]. Black boxes around RelA[hS] and SpoT[HS] residues show sites that have experienced shifts in substitution rate, as predicted with Diverge. C) Structure of the Rel[HS] protein from *S. equisimilis* (Rel_Seq_) [40], colored according to the conservation patterns of the alignment in B.

A consensus sequence alignment of Rel[HS], RelA[hS] and SpoT[HS] shows the domain structure of the long RSHs (Fig. 2A and 2B). With the exclusion of plant subgroups, most long RSHs have the full domain structure with the ACT and TGS domains (grey underlay, Fig. 1A and B). In addition to the known domains (HD, SYNTH, TGS and ACT), large blocks of conservation between the TGS and the ACT domain suggest two additional domains, separated by a predicted region of disorder of 16–36 amino acids in length in the consensus sequences. The first of these new domains is 88 amino acids long in consensus, and is predicted by Psipred to be composed entirely of helices. We therefore refer to it as the helical domain. The second new domain is 61 amino acids long in the consensus alignment and is predicted to be composed of four short sheets, followed by two short helices. Particularly striking in this domain is a conserved block that contains within it three conserved cysteines that are proposed to interact with the SYNTH domain [59]. We therefore call this domain the CC (conserved cysteine) domain. While most long RSHs carry the full six-domain structure, there are some rare exceptions. *Francisella tularensis* RelA[hS], *Francisella philomiraga* RelA[hS], *Methylotenera mobilitas* RelA[hS], and *Elusimicrobium minutum,* Rel[HS] lack the ACT domain. RshC[HS] is truncated within the helical domain, meaning it carries the TGS domain, but lacks most of the helical and all of the CC and ACT domains. The latter two domains are also missing from *Salinispora* RshA[HS]s. *Haemophilis ducreyi* and *Actinobacillus pleuropneumonia* SpoT[HS]s have an internal deletion, removing the helical and CC domains but keeping the ACT domain.

The consensus alignment of the RSHs allows the identification of differentially conserved sites indicative of a shift in functional constraints. As the crystal structure of the NTD (HD and SYNTH domains) of *S. equisimilis* Rel[HS] (“Rel_Seq_”) is available [40], the three-dimensional location of the NTD subset of these sites can be plotted (Fig. 2B and C). The NTD structure is roughly mussel shell-shaped, with a central three-helix bundle linking the SYNTH and HD nucleotide binding sites that are roughly symmetrical (Fig. 2C). The HD domain is mostly α-helical, forming a bundle, the base of which forms the nucleotide binding pocket along with an extended loop (residues 40–50 in Rel_Seq_ and positions 49–59 in Fig. 2B) [40]. Rel[HS] and SpoT[HS] are well conserved in residues that line the HD binding pocket, while RelA is clearly more divergent, consistent with its loss of hydrolase activity (yellow sites, Fig. 2B and C). RelA[hS] sequence divergence is most prominent in the N terminal half of the HD domain, which includes more nucleotide-interacting sites including the extended loop. The HD domain residues that are conserved in RelA[hS] as well as SpoT[HS]/Rel[HS] are oriented away from the binding pocket, and seem to interact with neighboring helices, possibly stabilizing the structure (Fig. 2C). A three-helix bundle forms the interface of the HD and SYNTH domains (Fig. 2C). Although these helices are within the Pfam HD domain model, the last two helices of the domain, and the loop between them contains a region that appears to form part of a pocket for GDP in the synthetase active site (positions 195–205 Fig. 2C) However, mutations in this region do not abolish synthetase activity [40], and the dispensability of this region for ppGpp synthesis is also suggested by its total absence in SASs.

In the SYNTH domain, many sites are conserved across Rel/RelA/SpoT (blue sites, Fig. 2B and C). Sites that are strongly conserved in RelA[hS] and differentially conserved for a different amino acid or weakly conserved in Rel[HS]/SpoT[HS] tend to be found more in the SYNTH domain, particularly the nucleotide binding pocket (green sites, Fig. 2B and C), than in the HD domain. This suggests there has been some refinement of the synthetase function in RelA[hS] following duplication of Rel[HS]. There is also more loss of conservation in SpoT[HS] in the SYNTH domain than in the HD domain: five sites are conserved in RelA[hS]+Rel[HS] that are unconserved in SpoT[HS] in the SYNTH domain, versus just one in the HD domain (purple sites, Fig. 2B and C).

Differences in functional constraints are also apparent in the CTD half of long RSHs. In the TGS domain, SpoT[HS] is just as different from Rel[HS] as is RelA[hS]. In particular, there is one sheet region that is enriched in RelA[hS]/Rel[HS] sites (“TGS variable region”, Fig. 2B). Additionally, the core of the ACT domain, i.e. the most strongly conserved region of the Pfam model, has greater conservation in RelA[hS] than in Rel[HS]/SpoT[HS]. As another indication of functionally divergent sites, independent of conservation, Diverge [53] was used to find those alignment positions where there has been a statistically significant shift in the amino acid substitution rate since the duplication of RelA[hS] and SpoT[HS]. Diverge ignores all sites containing gaps and so only samples a subset of sites in the whole alignment. However it identifies many sites that have a significant difference in rate between RelA[hS] and SpoT[HS] (black boxed residues, Fig. 2B). These functionally divergent sites are found in all domains of the protein, supporting observations from the consensus sequences that all domains contain sites under differential selection pressures. As also suggested by consensus sequences, the TGS variable region and the core of the ACT domain are hotspots for rate variation (Fig. 2B).

**Figure 3:**
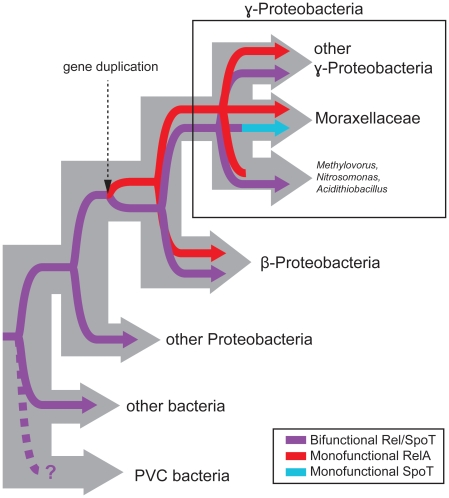
Schematic diagram for the evolution of long RSHs in bacteria. Thick gray branches indicate the divergence of bacterial groups, while the inner line shows the divergence of long RSH proteins and their functionality, as per the inset box.

Just as RelA[hS] has lost its HD function, some bacteria that encode both RelA[hS] and SpoT[HS] seem to be losing their SpoT synthetase function (SpoT[Hs]) (Fig. S2 and S3). The alignment of *E. coli, Acinetobacter* and *Psychrobacter* RelA[hS] and SpoT[HS]/[Hs] shows that while *E. coli* SpoT[HS] is conserved in important SYNTH sites, *Acinetobacter* and *Psychrobacter* (the Moraxellaceae [60]) SpoT[Hs]s are not (Fig. S3). Indeed, the SpoT[Hs]s of these organisms are so divergent, they have particularly long branches in the phylogenies and do not even group with the other γ-proteobacteria (Fig. 1B and S2). Thus, subfunctionalization of the synthetase and hydrolase functions of SpoT and RelA appears to be more “complete” in Moraxellaceae. Long RSH distributions suggest loss of RelA[hS] is rare, but possible; this subgroup was not identified in *Acidithiobacillus ferrooxidans*, *Candidatus Ruthia magnifica*, *Candidatus Vesicomyosocius okutanii*, *Methylovorus* SIP3, and *Nitrosomonas europaea,* (Fig. S2), suggesting four independent losses. In these cases, presumably their SpoT alone is sufficient as a bifunctional Rel[HS]. This pattern of gain and loss of long RSH SYNTH and HD functions allows us to hypothesize the evolutionary history of Rel[HS], RelA[hS], SpoT[HS] and their synthetase and hydrolase functions in different lineages of bacteria (Fig. 3).

### Phylogenetic and sequence analysis of long RSHs in plants

**Figure 4:**
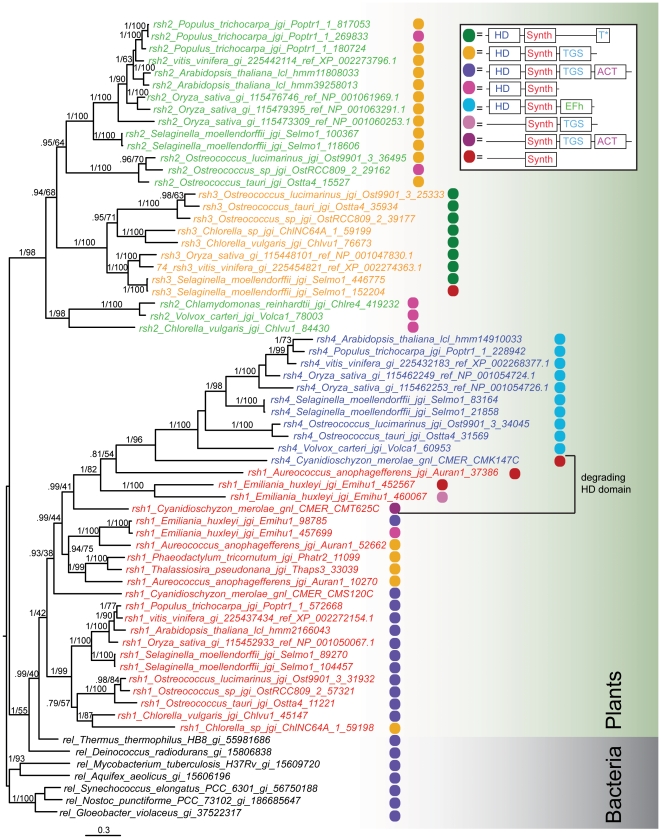
Bayesian inference phylogeny of plant RSHs. The tree was generated from a MrBayes analysis of 470 amino acid positions from 66 sequences. Colored sequence names indicate subgroups as follows: red – Rsh1, green – Rsh2, orange – Rsh3, blue – Rsh4, and black – bacterial Rel. Numbers on branches show support in the following format: BIPP/MLBP. Support is only shown for branches with BIPP>0.8. Branch length is proportional to the number of substitutions per site (see scale bar).

Four subgroups of ppGpp synthetases were found in plants: Rsh1-4 (Fig. S2). In the long RSH tree, the plant types group together with deinococci, although with weak support (44% MLBP, Fig. S2). To better resolve relationships in the plants and deinococci part of the tree, ML and BI phylogenetic analyses were carried out on a data set of plant sequences, plus deinococci, with other bacteria (*Mycobacterium*, *Aquifex* and cyanobacteria) as an outgroup (Fig. 4). In this tree, Rsh2[HS] + Rsh3[HS] are strongly supported as a monophyletic group (1.0 Bayesian inference posterior probability (BIPP), 98% MLBP), and deinococci Rel[HS] + Rsh1[HS] + Rsh4[HS] are strongly supported with BI (1.0 BIPP) but weakly supported with ML (55% MLBP) as a monophyletic group. Rsh2/3 and deinococci/Rsh1/Rsh4 appear as sister groups in the tree topology, but with no statistical support. The plant Rshs do not group with cyanobacteria, as would be expected for chloroplast genes (Fig. 4). However, a close relationship of cyanobacteria, deinococci and chloroplasts is seen with core conserved genes [61]. Thus, the failure of cyanobacteria to group with the other two in this case may be artifactual.

Rsh1[HS] has the widest distribution among plants and is found in various chromalveolates that inherited their chloroplasts from secondary endosymbiosis as well as archaeplastida and red algae (*Cyanidioschyzon merolae*) that inherited their chloroplasts through vertical descent [62] (Fig. 4). EF-hand domain-carrying Rsh4[HS]s, are monophyletic with full support (1.0 BIPP, 100 MLBP). A *C. merolae* RSH Rsh4[S] also groups with strong support (1.0 BIPP, 96% MLBP) with the other Rsh4s, although it does not carry an EF-hand domain, and its HD domain is degrading. Thus, the EF-hand domain may have fused to an Rsh1-like protein in the archaeplastida lineage after the divergence of *C. merolae*. The boundary between the Rsh1 and Rsh4 groups is uncertain due to the presence of divergent algal Rsh1/4[S]s with degrading HD domains. The chromalveolates *Aureococcus* and *Emiliania* have some long branched HD-degrading RSHs that cluster with Rsh4[HS] with reasonable support (1.0 BIPP, 82% MLBP), suggesting these may have also originated from the Rsh duplication that gave rise to Rsh4[HS], before the secondary endosymbiosis event (Fig. 4). Additionally, as *C. merolae* has Rsh1[HS], Rsh1[S] and Rsh4[S], multiple duplications appear to have been involved. Rsh2[HS] and Rsh3[HS] are only found in green plants (both land plants and algae) and there is strong support for the monophyly of these groups together (1.0 BIPP, 98% MLBP, Fig. 4). Rsh2[HS] is not supported as monophyletic, however, Rsh3[HS] has full support for monophly (1.0 BIPP, 100 MLBP).

Rshs have previously been identified in Arabidopsis and named Rsh1-4 [63]. However, the Arabidopsis-named Rsh2 and Rsh3, are both in fact recent duplications of Rsh2[HS], and a true Rsh3[HS] is actually missing from Arabidopsis (Fig. 4). Rsh3[HS] is however found in other multicellular plants, so appears to have been lost in the lineage to Arabidopsis.

The consensus alignment of plant sequences shows the differences in conserved length among each plant RSH (Fig. S4). In terms of domain structure, plant long RSHs are much more diverse than those of bacteria (Fig. 1A, 1B and 4). Only Rsh1[HS] is the full length form equivalent to Rel/RelA/SpoT, with the C terminal ACT, TGS and helical domains, although with the CC domain unconserved (Fig. S4). Rsh2[HS] and Rsh3[HS] also have the TGS domain, which is lacking in Rsh4[HS], having acquired the EF-hand domain at its C terminus. Rsh3[HS] sequences have an insertion in between the SYNTH domain and the TGS domain, the latter of which has only a fragment remaining at the extreme C terminus of Rsh3[HS] (referred to here as the T* domain, Fig. 1A–B and 4). The degradation of the HD domain in some plant RSHs suggests subfunctionalization is occurring within plants similarly to the subfunctionalization of RelA[hS] and SpoT[HS] in bacteria (Fig. 4).

### Phylogenetic analysis of small RSHs and comparison of their sequences with Long RSHs

In comparison to the long RSHs, the taxonomic distributions of the SASs and SAHs are in general sporadic, with their presence and absence spread across the diversity of RSH-encoding bacteria (Table 1). Among our 12 subgroups of SASs, only three have been previsously reported: RelV[S], RelQ[S] and RelP[S] [27], [29]. The SASs and SAHs are in general small, single domain proteins. An exception is the actRel subgroup of SASs, within which some members (*Mycobacterium gilvum*, *Mycobacterium smegmatis*, *Mycobacterium vanbaalenii*) carry an N terminal domain of unknown function (“DUF429” in Pfam). DUF429 usually occurs on its own as a single domain protein in bacteria and archaea, but in a handful of bacteria, it is found in combination with other domains involved in nucleoside metabolism: the “Nudix” hydrolysis of nucleoside diphosphates domain, and the phosphomethylpyrimidine kinase domain, which is a phosphotransferase of the thiamine pyrophosphate (TPP) synthesis pathway.

**Figure 5:**
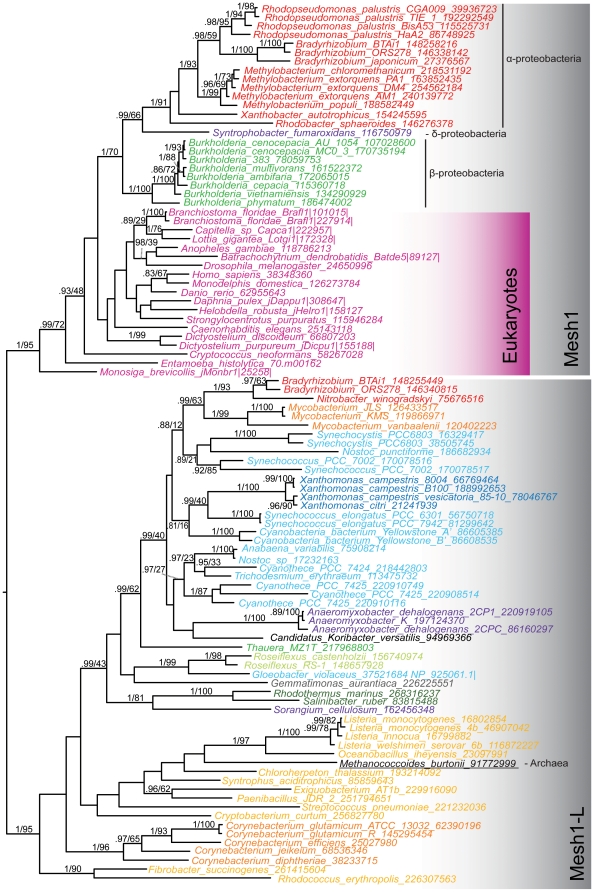
Bayesian inference phylogeny of the SAHs Mesh1 and Mesh1L. The tree was generated from a MrBayes analysis of 179 amino acid positions from 99 sequences. Branch support and length are shown as described in Fig. 4. Sequence names are colored by taxonomic groups.

In eukaryotes, the only widely spread subgroup is Mesh1[H]. This is also the only previously reported SAH for any organism; surprisingly, no bacterial SAHs have been reported before the seven subgroups we describe here. Phylogenetic analysis of the Mesh1[H] and Mesh1-L[H] subgroups show eukaryotic Mesh1 groups tightly with a clade of α-, β- and δ-proteobacteria (1.0 BIPP, 95 MLBP, Fig. 5). Mesh1 could be identified in the genomes of animals, amoebozoa, the choanoflagellate *Monosiga brevicollis* and the fungus *Cryptococcus neoformans*. It was, however not identified in any other fungi. Scans of eukaryotic Mesh1[H]s on the TargetP server [55] show no mitochondrial target peptides, suggesting they are cytoplasmic proteins. Mesh1-L[H] is the sister group to Mesh1[H] (Fig. 1A), and as Mesh1[H] and Mesh1-L[H] taxonomic distributions are largely non-overlapping, these two clades may in fact be orthologous. However, there is no statistical support for the monophyly of the two groups (Fig. 1A). Additionally, HGT appears to have played a considerable role in Mesh1/Mesh1-L[H] evolution. In particular, the messy phylogeny of Mesh1-L[H] suggests it has been transferred multiple times (Fig. 5). Mesh1-L[H] was also found in the genome of *Methanococcoides burtonii*, making it one of the few RSHs to be found in archaea.

Although several genera of α-proteobacteria carry Mesh1[H], the Rickettsiales do not. Rickettsiales have their own group of hydrolases (rickSpo[H]), which in some lineages has experienced extensive proliferation, associated with transposable elements [64]. The most extreme case is *Orientia tsutsugamushi* (strain Ikeda), in which 40 sequences matched the rickSpo[H] HMM (Tables 2, S1 and S2) [65]. In *Rickettsia felis*, all of its 14 RSHs are transcribed (Tables S1 and S2) [66], and in *Rickettsia conorii*, its five RSHs are differentially transcribed, depending on environmental conditions [67]. There is also a Rickettsiale-specific group of synthetases (rickRel[S]), which has previously been found to be involved in virulence [68]. Some rickRels also carry the HD domain, although it is very divergent (rickRel[hS], Fig. 1A and B). A subset of the rickRel[S] encoding taxa also encode rickSpo[H], which phylogenetic analysis shows is not an ortholog of the HD domain of rickRel[hS] (Fig. 1A). Therefore, these groups appear to have arisen independently, rather than from the splitting of one Rel[HS] or rickRel[hS] ortholog. Indeed, *O. tsutsugamushi* also encodes a full length Rel[HS] ortholog.

Mesh1[H] and plant RSHs are the main, but not only RSHs in eukaryotes. Divergent SASs that could not be assigned to any subgroup (divRel[S]s) were found in the fungi *Aspergillus nidulans*, *Aspergillus fumigatus*, *Gibberella zeae*, in the amoebae *Dictyostelium discoideum* and *Dictyostelium purpureum,* and in the heterokont algae *Thalassiosira pseudonana* and *Phaeodactylum tricornutum*. Eukaryotes appear able to carry SAHs without SASs and vice versa. While the dictyostelid amoebae encode Mesh1[H], and the algae encode Rsh1[HS] (plus a divergent Rel[HS] subgroup member in the case of *T. pseudonana*) as hydrolases, the fungi do not carry an RSH hydrolase. Similarly, one divRel[S] was found in the archaeon *Methanosarcina acetivorans*, also lacking an SAH. SAHs were found however in the archaeon *Methanosarcina barkeri* (divSpo[H]), *Methanococcoides burtonii* (Mesh1-L[H]), *Natronomonas pharaonis* (pbcSpo[H]) and uncultured methanogenic archaeon RC-1 (pbcSpo[H]), none of which carry an SAS (Tables 2, S1 and S2). The polychaete annelid worm *Capitella Sp.* has three predicted RSHs: Mesh1[H], RelA[hS] and SpoT[HS]. However, the RelA and SpoT sequences are very similar to *Pseudomonas* sequences and are encoded on short scaffolds, suggesting these are cases of genome sequence contamination.

**Figure 6:**
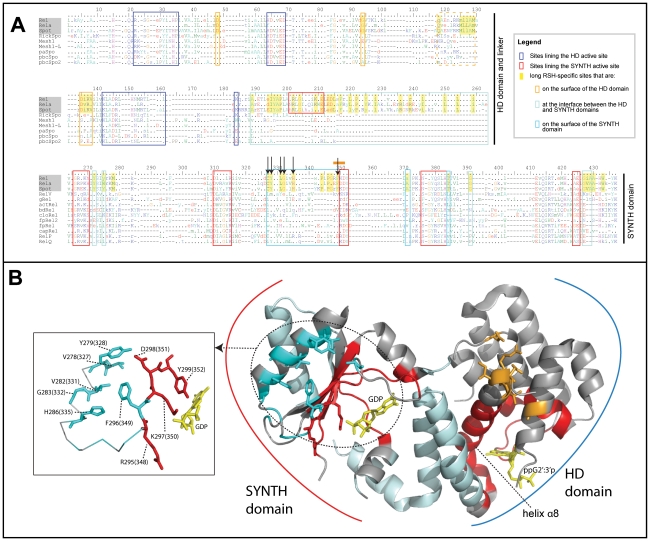
Consensus alignment of long RSH and small RSH subgroups across the ppGpp synthetase and hydrolase domains, with Rel_Seq_ NTD structure colored according to conservation patterns in the alignment. A) Alignment of RSH NTD sequences at the 70% level. Yellow highlighting shows those residues that are only conserved only in long RSHs. Blue and red boxes show sites of the HD and SYNTH nucleotide binding pockets, respectively. Bright turquoise and orange boxes show the location of surface residues in the SYNTH and HD domains respectively that are likely to be involved in inter molecular interactions, or interactions with the CTD in long RSHs. The box is dotted where the region is disordered in the structure. The pale marine box shows those regions that appear to be involved in HD-SYNTH interactions. Arrows show especially interesting sites (see inset box in B). The orange bar above the alignment shows the location of the differentially conserved motif of [44]. B) Structure of the Rel[HS] protein from *S. equisimilis* (Rel_Seq_) [40], colored according to the conservation patterns of the alignment in A. The inset box shows a subset of particularly interesting sites (labeled with arrows in A). Residue numbering is as in Rel_Seq_, followed by alignment coordinates from A in parentheses.

Residues conserved in both the long and small RSHs indicate the required core of the SYNTH and HD domains (Fig. 6A and B). The C terminal domains of Rel[HS] have been proposed to interact with and regulate the SYNTH domain [43], [59], however it is not known which residues in the SYNTH domain are responsible for transmitting signals from the CTD. As signal transmitting sites would likely only be conserved in CTD-containing long RSHs, and not CTD-lacking SASs, comparative analyses of sequence conservation in combination with the X-ray structure of the NTD region allows us to predict molecular interacting sites specific to the long RSHs. Such interacting sites may be involved in NTD to CTD intra-molecular interactions, and/or inter-molecular interactions among individual RSH molecules or with long RSH binding partners such as the ribosome. A consensus sequence alignment of just the SYNTH and HD domains shows the sites that are limited in conservation to the long RSHs Rel[HS], RelA[hS] and SpoT[HS] (yellow highlighting, Fig. 6A and B). Most long RSH-only sites are found in the helices linking the SYNTH and HD domains, which in unsurprising as small RSHs do not require this linker region (Fig. 6B). In the HD domain, 12 long RSH-specific residues are located on the surface of the protein (Fig. 6A and B, Table 3), including those in an exposed flexible loop disordered in the structure. The exposure of these sites suggests they are involved in inter- or intra- molecular interactions, which could potentially alter the conformation of the nucleotide-interacting helix α8 (Fig. 6A and B). On the surface of the SYNTH domain, there are 11 exposed well conserved long RSH-only sites that could be involved in long RSH-specific inter- or intra- molecular interactions (Fig. 6A and B, Table 3). Of these, six appear to directly interact with the SYNTH active site (bold highlighting Table 3, Fig. 6A and inset box, Fig. 6B) In the numbering of alignment Fig. 6A, these are C327 (Val in *Streptomyces*), Y328, L331, G332, H335 and F345. These residues overlap with a differentially conserved motif of RelA[hS] and SpoT[HS]; RelA[hS] has a conserved acidic motif (EFDD) that is differentially conserved as basic RFKD in Rel[HS]/SpoT[HS] (348–351, Fig. 6A [24]). These have been suggested to give rise to a preference for GDP and GTP in the SYNTH domains of RelA[hS] and Rel[HS]/SpoT[HS] respectively [44]. The Phe (F296 in Rel_Seq_ and F349 in Fig. 6A) of the motif is unconserved in SASs, although conservation is seen of the downstream “DY”, which is positioned inside the nucleotide binding pocket (Red residues, inset box, Fig. 6B). The orientation of the Phe residue potentially makes it capable of interacting with the other long RSH-only residues (inset box, Fig. 6B), suggesting this is a critical residue for signal transmission from the CTD or other molecules to the SYNTH active site.

**Table 3:** Long RSH-specific sites that may be involved in intra- or inter-molecular interactions in long RSHs.

Predicted interactions	Rel_Seq_ residue	Rel_Seq_ residue number	*E. coli* RelA residue	*E. coli* RelA residue number	*E. coli* SpoT residue	*E. coli* SpoT residue number	Fig. 2B numbering	Fig. 6A numbering
HD-X interactions	D	67	D	72	D	62	76	48
	G	104	G	109	G	99	103	94
	*L*	*120*	*Q*	*131*	*Q*	*115*	*138*	*118*
	*MLMAM*	*127–131*	*MLLAM*	*138–142*	*MIMAM*	*122–126*	*145–149*	*125–129*
	DIRV	134–137	DFRC	145–148	DIRV	129–132	152–155	145–148
SYNTH-HD interactions	S	164	A	175	A	159	183	189
	EIYAP	169–173	NIYAP	180–184	EIYSP	164–167	188–192	194–198
	R	177	R	188	R	172	196	202
	G	179	G	190	G	174	198	204
	K	184	K	195	K	179	203	209
	E	186	E	197	E	181	205	211
	EDL	188–190	EDY	199–201	EEL	183–185	207–209	213–215
	F	192	F	203	F	187	211	217
	L	194	L	206	L	190	214	220
	E	197	P	208	P	192	216	222
	F	200	Y	211	Y	195	219	225
	Y	246	Y	257	Y	241	266	272
	Y	249	W	260	Y	244	269	275
SYNTH-X interactions	**VY**	**278–279**	**CY**	**289–290**	**CY**	**273–274**	**298–299**	**327–328**
	**VG**	**282–283**	**LG**	**293–294**	**LG**	**277–278**	**302–303**	**331–332**
	**H**	**286**	**H**	**297**	**H**	**281**	**356**	**335**
	P	291	H	302	P	286	311	344
	PG	293–294	PD	304–305	PG	288–289	313–314	346–347
	**F**	**296**	**F**	**307**	**V**	**291**	**316**	**349**
	A	301	A	312	A	296	321	371
	T	313	T	324	T	308	333	384
SYNTH-HD interactions	G	317	G	328	G	312	337	390
	GVA*A*	338–341	GVA*A*	350–353	GVA*A*	334–337	359–*362*	427–*430*
CTD-NTD interactions	CC	608–609	CC	612–613	CC	573–574	629–630	N/A
	P	611	P	615	P	576	632	N/A
	P	613	P	617	P	578	634	N/A
	D	615	D	619	D	580	636	N/A
	I	617	I	621	I	582	638	N/A
	G	624	G	628	G	589	645	N/A
	G	626	G	630	G	591	647	N/A
	H	630	H	634	H	595	651	N/A
	C	634	C	638	C	599	655	N/A

In the predicted interactions column, “X” indicates either the CTD or another interacting molecule. Residues in italics are disordered in the structure and those in bold are sites of the inset box in Fig. 2B.

## Discussion

### RelA subgroups, distribution and organization of the RSH systems

We have classified the RSHs into a total of 30 subgroups of RSH proteins, compared with four (two classes of Rel divided by taxonomy, plus RelA and SpoT) that were identified in the previous evolutionary analysis of this protein superfamily [3]. Here, we treat Rel[HS] as one subgroup, as we attempt to classify apparently orthologous proteins into the same subgroup. Our categorization allows us to retrace the evolutionary history of subgroups of RSH proteins and raises questions about the composition and organizational principles of RSH-mediated stringent response networks. By analyzing possible combinations of RSH proteins and their domains in bacterial genomes, and comparing RSH content with available RSH knock-out data for various organisms [18], [28], [68], we attempt to interpret the functional consequences of RSH gain and loss and rationalize the design rules of RSH sensory systems [69].

Bifunctional Rel[HS] is by far the most widely distributed of the RSH proteins, being found in all phyla except members of the PVC superphylum, β- and γ-proteobacteria, the latter two of which encode the Rel[HS] duplicates RelA[hS] and SpoT[HS] (36 of 41 phyla, Tables 1 and 2). Rel[HS] was either lost or was never present in PVC bacteria, depending on the relationships among PVC and other major groups of bacteria, which still remains elusive [70]. Lack of long RSHs is one more feature that members of the PVC superphylum have in common with eukaryotes and archaea, along with attributes such as a compartmentalized cell plan, loss of peptoglycan and loss of the FtsZ cell division protein [71].

Other bacteria that lack RSHs are mostly obligate intracellular parasites or endosymbionts, as previously reported [3]. Of free-living organisms outside of the PVC superphylum, RSHs could not be found in seven species of *Mycoplasma*, three species of spirochetes, and *Thermoanaerobacter* X514 of Clostridiales. Most of these organisms are pathogenic (*Treponema pallidum* and *Brachyspira hyodysenteriae* of the spirochetes, and all the species of *Mycoplasma*). However, all these taxa have close relatives that are also pathogenic but carry RSHs. For example, *Mycoplasma genitalium*, which has one of the smallest genomes of free-living organisms, carries Rel[HS]. Thus, loss of the RSH system is possible in pathogenic bacteria, but rare, unless the bacterium is an obligate intracellular parasite.

The co-distribution of RelA[hS] and SpoT[HS] in γ- and β-proteobacteria that lack Rel[HS] suggest they originate from a duplication of Rel[HS] in the proteobacterial lineage after the divergence of α-proteobacteria, the most closely related class to β- and γ-proteobacteria. The advantage of having a rapid ppGpp on/off switch may have driven the subfunctionalization of RelA[hS] and SpoT[HS] into mostly synthetic (RelA[hS]) or hydrolytic (SpoT[HS]) proteins following duplication of the ancestral Rel[HS]. In bacteria that encode RelA[hS] and SpoT[HS], concentrations of the synthetase and hydrolase domains are not kept equal as in the case of a single bifunctional Rel[HS] protein.

The ppGpp-mediated regulatory circuits just as any sensory network, are challenged by noise originating from the stochastic nature of the chemical reactions constituting life [72], [73]. This noise can be countered by wiring enzymatic networks that are insensitive to fluctuations in the protein concentrations [74], [75], or alternatively can be used to create phenotypic heterogeneity to diversify the population [76]. In the case of Rel[HS], heterogeneity in expression has been recently documented in *Mycobacteria*, where noise in Rel[HS] expression is further amplified via a positive loop acting on the *mprAB* operon [77], suggesting that cellular diversification, rather than system stability is the strategy in this case. This observation, combined with the well-documented role of ppGpp in bacterial survival and virulence [78], [79], [80], is consistent with the documented role of phenotypic heterogeneity in the persisting survival of a subset of a bacterial population (so called persistor cells) following treatment with antimicrobials [81]. In organisms with multiple RSHs, heterogeneity is potentially much more significant due to independent regulation and stochastic noise in the expression levels of different RSH proteins, which, in turn, will lead to phenotypic heterogeneity essential for persistence in adverse environmental conditions [82].

Apart from the potential kinetic advantages of encoding synthetic and hydrolytic activities in different polypeptides, the subfunctionalization of RelA[hS] and SpoT[HS] also allows bacteria to sense different environmental cues through different intermolecular interactions. SpoT[HS] in *E. coli* is known to have interaction partners distinct from RelA[hS], such as the acyl carrier protein [9] (and see below). The functional diversification of SpoT[HS] and RelA[hS] is evident in the patterns of amino acid substitutions across the proteins (Fig. 2B). Comparative analysis of these subgroups indicates functionally divergent sites (sites with differential conservation and/or substitution rate shifts) are not limited to the hydrolysis and synthetase domain but are distributed over the full lengths of the proteins (Fig. 2B).

The expansion of SASs and SAHs possibly fine-tunes sensitivity and speed of reactions to the “classical” stringent response cues: different SASs and SAHs can be transcribed from different promoters, adding a transcriptional level of regulation to stringent response machinery. The SASs and SAHs may be expressed in response to different environmental triggers, so acting synergistically. Independent expression of long and small RSHs from multiple genes may also be beneficial to bacteria on a population level. In *E. coli*, intracellular ppGpp concentration is the primary factor controlling growth rate [37]. As random fluctuations are intrinsic to gene expression [83], cell-to-cell variability in RSH expression can result in heterogeneity in sensitivity to the stringent response signal within the population.

The core RSH set can accommodate addition of various combinations of SASs and SAHs. While 92% of the bacterial genomes sampled here carry Rel[HS] or SpoT[HS], just 44% of bacteria have Rel[HS] alone, SpoT[HS] alone, or RelA[hS] plus SpoT[HS] without any other RSHs. The SASs and SAHs have very scattered distributions across bacteria, with multiple subgroups often present in the same organism, and with the RSH complement often differing widely within families of bacteria, and sometimes within genera (Table S2). This suggests that HGT has played a major role in their evolution. In contrast, the core long RSHs seem to have a low propensity for HGT. RelA[hS] or SpoT[HS] are never found in Rel[HS]-encoding organisms, or vice-versa, and most major groups of bacteria form clades in the long RSH tree indicating mostly vertical descent (Figure S2). Of the few long RSH subgroups that represent extra copies of Rel[HS] in one genome, only RshC[HS] and RshD[HS] (ten taxa in total) are not clearly derived from gene duplication, and are candidates for possible horizontal origin, although from unknown donors. The horizontal mobility of the SASs and SAHs is probably promoted by their being simple “stand-alone” single domain modules, that presumably have few intermolecular interactions as compared to the long RSHs. The long RSHs have many interactions with other molecules that regulate their activity (see below). Thus, under the complexity hypothesis, this complicated network of interactions would make them less prone to HGT [84].

The analysis of naturally occurring RSH systems in bacteria suggests certain organizational rules. Firstly, no bacteria with RelA[hS] or an SAS alone are observed, indicating that loss of the hydrolytic component (SpoT[HS] or SAH) is prohibited, presumably because that would result in run-away responses leading to cell death. Secondly, loss of the major synthetic component, is rare but possible, with several organisms (*Acidithiobacillus ferrooxidans*, *Candidatus Ruthia magnifica*, *Candidatus Vesicomyosocius okutanii*, *Methylovorus* SIP3, and *Nitrosomonas europaea*) having only SpoT[HS] and no RelA[hS]. SpoT[HS] has some synthesis ability, explaining its presence without RelA[hS], however, it is also possible (although rare) for an SAH to be present alone in bacteria (*Rickettsia conorii*). Thus, although we have identified more SAS than SAH subgroups, the distribution of the HD domain is wider than the SYNTH domain. Knock-out experiments in *E. coli* corroborate well with our observations: strains with knock-outs of RelA[hS] or simultaneous knock-out of RelA[hS] and SpoT[HS] are viable, whereas knock-out of SpoT[HS] is lethal [18]. Reported *ΔspoT* strains [85] have compensatory mutations in RelA[hS], which compromise its synthetic activity (see erratum for that paper). Similarly, SASs can be toxic when expressed alone without an RSH ppGpp hydrolase in the cell [28]. This suggests that HGT of an SAS is probably more likely to be successful if the host already contains a dedicated SAH for ppGpp hydrolysis. *Treponema denticola* is the only identified case of a bacterium that encodes no Rel[HS], but has SAS and SAH proteins that seem to have originated from HGT.

### RSHs in eukaryotes and archaea

Among eukaryotes, RSHs are most widespread in plants, which have four RSH subgroups (Fig. 4). These are likely to have originated by gene duplication, and seem to have diversified in function through loss of domains in some subgroups and gain of the calcium binding EF-hand domain in the case of Rsh4[HS] [86] (Fig. 4 and S4). Plant RSHs are much more varied in terms of domain architecture than bacterial long RSHs, indicating much greater flexibility is allowed in terms of long RSH domain complement. However, in plants, as is the case in most bacteria, a hydrolase is not present without a synthetase and vice-versa.

Animals have been found to carry Mesh1[H], an SAH [30]. We find Mesh1[H] in various opisthokonts: animals, amoebae and one fungus (*Cryptococcus neoformans*, Fig. 5). Mesh1[H] is also found in some (but not all) proteobacteria, including α-, γ- and δ proteobacteria. There are three possibilities for the origin of Mesh1[H] in opisthokonts: firstly, it may be the ortholog of bacterial RSHs, and was inherited by eukaryotes through vertical descent. However, this implies that archaea also carried Mesh1[H], which was lost almost universally across archaea, in addition to much loss in eukaryotes. We only find one Mesh1-like (Mesh1-L[H]) protein in an archaeon, *Methanococcoides burtonii*. A more parsimonious explanation that requires less loss is that Mesh1 entered eukaryotes with the ancestor of the mitochondrion. However, this also requires loss in all eukaryotes except the opisthokonts and there is no evidence in the form of mitochondrial localization or transit peptides that Mesh1[H] is or was an organellar protein. Finally, as HGT from bacteria directly into eukaryotes is relatively common, especially for single celled eukaryotes (as was the ancestor of opisthokonts) [87], gene transfer seems to be the simplest hypothesis for the origin of Mesh1[H].

Similarly, the most likely explanation for the sporadic distribution of divRel[S] SASs in eukaryotes and archaea is HGT from bacteria. Phylogenetic relationships that might support possible donors of these RSHs unfortunately lack statistical significance. However, it is interesting to note that all of the recipient eukaryotes spend all or part of their life cycles in the soil (*Dictyostelium*, *Aspergillus and Gibberella),* suggesting SASs may be particularly useful to eukaryotes in this environment, possibly by producing ppGpp as an extracellular nucleotide signal, similar to the use of cAMP in *Dictyostelium* [88]. The production of ppGpp by *Dictyostelium* has previously been reported, although others have failed to repeat this finding [89] (and references therein).

The eukaryotic and archaeal RSHs do not comply with the rule in bacteria that a SAS cannot be present without an SAH. Surprisingly, SAHs were not identified in the genomes of SAS-encoding *M. acetivorans*, *Dictyostelium*, *Aspergillus and Gibberella.* It is possible that non-homologous or very divergent hydrolases are responsible for ppGpp in these organisms. Such a hydrolase has been identified in the bacterium *Thermus thermophilus,* which in addition to its Rel[HS] encodes a ppGpp-degrading Nudix pyrophosphatase, a member of a protein family with homologs across the tree of life [90].

SAHs can also occur alone in eukaryotes. Although the SAH Mesh1[H] is widespread in animals, our search did not identify any SASs in this kingdom. Similarly, five euryarchaea (*M. barkeri, M. burtonii, N. pharaonis* and uncultured methanogenic archaeon RC-1) have SAHs apparently without SASs. These organisms may encode non-homologous ppGpp synthetases, as previously suggested [30]. However no non-RSH proteins are currently known to produce ppGpp. The only previously reported non-homologous ppGpp synthetase is the secreted ppGpp synthetase of *Streptomyces morookaensis* [12], [91], which our HMMs show is in fact a divRel[S] SAS with a significant E value of 8E^−7^ for the presence of the SYNTH domain.

### RSH domain organization and interaction partners

Long RSH proteins are known to interact with numerous ligands, including the ribosome [11], tRNA [59], ppGpp in an allosteric site [59] and their substrates ATP and GDP[11]. SpoT additionally interacts with ppGpp in its hydrolytic site [19], the acyl carrier protein ACP [9] and the Obg/Gtc GTPase [92]. However, the scarcity of high-resolution structural data (especially the absence of crystal structures for complexes or cryoEM reconstructions of the ribosome-bond RSH) hinders domain- or site-specific prediction of function. Even tRNA cross linking to discover the tRNA binding site is not applicable for RelA since this protein does not interact with CCA-modified tRNAs [93]. Most of the available data mapping RSH interactions come from *in vivo* experiments using truncated proteins[ [9], [26], [41], [94], [95] where proper folds and, therefore functions are hard to verify. Thus, the function of some domains is obscure (the CC, helical and ACT domains) or there are contradicting results regarding their role (TGS) [96], [97].

Another issue that prevents successful mapping of sequence variation patterns with different RSH interactions is the promiscuity of protein/protein interactions in the same domain. Within the same protein, one interface can be involved in different interactions in different proteins, precluding one-to-one structure-function analysis [98]. However, despite the lack of specific functional information available at present, our identification of the helical and CC domains, additional potential conserved domains in the CTD, enables functional interactions to be explored in future experiments through targeting of these particular domains (Fig. 2B).

### Cis and trans interactions in long RSHs

RSHs have been shown to regulate their catalytic activity by interacting with themselves *in cis* via inter-domain cross-talk [43], [59] and in *trans* via oligomerization [41], [42], [59], [95]. In these complexes, Rel[HS] molecules have a low affinity for each other, as can be inferred from the low abundance of the complex when subjected to gel filtration analysis [41]. Taking into account the low *in vivo* concentration of RelA[hS] (50–100 nM) [99], it could well be that the observed dimerization is an artifact caused by the unnaturally high concentration of long RSHs in the *in vitro* system. When long RSHs are in close proximity, *in cis* cross talk may occur *in trans* through the interaction of the NTD of one protein molecule with the CTD of another. Thus, it is unclear whether, or just how significantly, oligomerization occurs *in vivo*.

Our hypothesis that oligomerization is a consequence of inter-domain cross talk is supported by the overlap in residues involved in both the *in trans* and *in cis* interactions in RSHs. Both types of interactions seem to be mediated by the same conserved Cys residues [59]. In *E. coli* RelA[hS], C612, D637, and C638 of the CC domain are involved in dimerization, with mutations of D637, and C638 residues negatively affecting ppGpp synthetic activity [41], [42].

Single-domain RSHs are not capable of inter-domain cross-talk, thus their sequences are expected to lack the conserved residues of long RSHs that mediate these interactions in the NTD region. On the basis of differential conservation patterns, we propose 23 residues that are potentially involved in mediating inter-domain cross-talk in the long RSHs. These are 11 residues of the SYNTH domain and 12 of the HD domain (Table 3). Of the 11 long RSH-specific sites in the SYNTH domain, we predict C327, Y328, L331, G332, H335, F345 (Fig. 6A and B) are critical residues for signal transduction to the SYNTH active site. These residues overlap with the RXKD motif at position 348–351 (Fig. 6A and B), which has a role in the interactions among long RSHs and their synthesis substrates (GTP versus GDP). For Rel[HS] from *Mycobacterium tuberculosis* (“Rel_Mtb_”) and RelA[hS] from *E. coli,* this motif was shown to confer G nucleotide phosphorylation state specificity, with EXDD (in RelA) and RXKD (in Rel[HS] and SpoT[HS]) conferring GDP and GTP preference, respectively [44]. These motifs are conserved only in the long RSHs (Fig. 6A), which reinforces the link between nucleotide specificity and the intra-molecular regulation of RSH activity. Additionally, the RelA[hS] versus SpoT[HS] differential conservation of these sites suggests that there are differences not only in nucleotide specificity, but also in how signals from the CTD are transmitted to the SYNTH active site in RelA[hS] as compared with Rel[HS]/SpoT[HS].

### Subfunctionalization of RelA[hS] and SpoT[HS]

Gene duplication results in a redundant copy of the original gene that is free from selective pressure and able to diverge in sequence [100]. Although the accumulation of mutations usually results in one copy being pseudogenized and lost, in some cases patterns of substitution result in both copies being maintained. If the fixation of the duplicates is a neutral process, their fate can be described by three models: neofunctionalization, duplication–degeneration–complementation (DDC) and specialization (reviewed in [101]). In the neofunctionalization model, one copy survives by adopting a new role distinct from that of the ancestral gene, while in the DDC model, facets of the ancestral function are partitioned between the duplicates (subfunctionalization). Specialization is a particular type of subfunctionalization, where duplication allows refinement of some functional features that was not possible in the original protein due to constraints imposed by dual functionality.

In the Rel[HS], RelA[hS] and SpoT[HS] system, we see three main fates of the genes and their synthesis and hydrolysis functions following duplication (Fig. 3). The first is loss of one of the duplicates, which we see in seven cases (corresponding to four independent lineages) which lack RelA[hS] (Figure S2). The second is complete subfunctionalization of hydrolysis and synthesis functions into SpoT[Hs] and RelA[hS] respectively, as is seen in the Moraxellaceae γ-proteobacteria. The third and most common fate is maintenance of the dual synthesis/hydrolysis function of SpoT[HS], but loss of hydrolysis function in RelA[hS]. This asymmetric pattern does not fit with the DDC model of subfunctionalization, as it does not explain retention of RelA[hS] in organisms with bifunctional SpoT[HS]. Similarly, at the sequence level, we do not observe a simple loss of conservation in RelA and SpoT[HS] as is expected under the DDC model [101] (Fig. 2B). This suggests asymmetric specialization more accurately explains the pattern we observe, with SpoT[HS] taking on most of the ancestral Rel[HS] function, while RelA[hS] loses some aspects of function and refines others.

Such refinement of RelA[hS] may involve its ppGpp synthesis function. The presence of sites that are strongly conserved in the RelA[hS] SYNTH domain and differentially conserved for a different amino acid or weakly conserved in Rel[HS]/SpoT[HS] suggests there has been some evolutionary fine-tuning of the synthetase domain in RelA[hS]. Specialization of RelA[hS] and SpoT[HS] probably also extends into the CTD: there are differentially conserved and rate shifting sites in all domains of the CTD (Fig. 2B), and differences in function have been found in experimental work. For example, SpoT[HS] alone has evolved a specific stress response role that involves interactions with ACP[9]. Neofunctionalization could also be occurring in the CTDs, which is, however, difficult to confirm from sequence alone due to unknown boundaries and likely overlapping of interaction sites. However, one case of neofunctionalization of RSHs is clear in plants, where Rsh4[HS], has acquired a new, calcium binding domain [86].

The loss of hydrolysis function in RelA[hS] raises the question of why the HD domain has not been lost in its entirety in this protein. Although poorly conserved, the supposedly non-functional HD domain of RelA[hS] has been maintained at least structurally throughout the evolution of β- and γ-proteobacteria. As the sites that are conserved in RelA[hS] in the HD domain appear to be important for structural integrity (Fig. 2C), this indicates that in all the full length RSHs, the HD structure is required for some purpose, in addition to its hydrolysis role, perhaps stabilizing the SYNTH domain, transducing signals from the CTD, or intermolecular interactions.

### Conclusions and outlook

The current analysis classifies RSHs into 30 RSH subgroups across the tree of life, 19 of which were previously unreported. These include previously unknown RSHs in archaea, fungi and Dictyostelid amoebae in addition to new bacterial RSHs with unusual domain structures. All these proteins now can be subjected to follow-up experimental analysis. The classification introduced in this study provides a unifying nomenclature for the RSH superfamily, resolving terminological confusion within the field (Table 1). We suggest that in the future, newly identified RSH genes that are not already present in Table S1 should be assigned to an RSH subgroup on the basis of phylogenetic analysis along with RSHs of known classification (Table S1), or by scanning the sequence with the subgroup-specific HMMs, available from us on request.

The wide variety of SAS and SAH combinations that are present in bacterial genomes leads us to hypothesize that the small RSHs are accessory genes that are easily gained and lost during evolution, allowing bacteria to dynamically rewire and refine their stress responses in a lineage-specific manner. The long RSH component on the other hand represents a core ribosome-interacting hub that has evolved conservatively since its origin early in bacterial evolution, maintaining its six-domain structure and specializing some aspects of its function on a conserved structural frame. Systems biology investigations of different RSH systems similar to that undertaken for *M. tuberculosis* Rel[HS] (“Rel_Mtb_”) [77] are needed to dissect the design principles of the stringent response system. We have discovered long RSH-specific residues on the surface of the SYNTH and HD domains that we predict are involved in interactions that regulate long RSH function. These residues are prime targets for site-directed mutagenesis to establish the role of molecular interactions in long RSH function.

The prolonged conservation of the long RSHs RelA[hS] SpoT[HS] and Rel[HS] indicates their importance in the cell, however there is still much that remains unknown about their function. In order to understand how the long RSHs interact with their binding partners, we need more structural data such as crosslinking, cryoEM and X-ray structures of RSH complexes, as well as full length RSH proteins themselves. This information in combination with biochemical and *in vivo* experiments will allow the wealth of the evolutionary and sequence data we have collated to be used to link sequence, structure and function.

## Supporting Information

Figure S1 **Phylogeny generated by DIVERGE for the analysis of site-specific rate shifts.** RelA and SpoT are shown in pink and blue, respectively.
(PDF)

Figure S2 **Maximum Likelihood phylogeny of the long RSHs using all alignable domains.** The tree is generated from 699 amino acid positions. Bootstrap support values greater than 70% are shown on branches. All subgroups are labeled except for the paraphyletic Rel, which is shown in purple. Major taxonomic groups are indicated on the right.
(PDF)

Figure S3 **Sequence alignment of RelA[hS] and SpoT[HS]/[Hs] from Escherichia coli, Psychrobacter arcticum and Acinetobacter baumannii.** Domains are indicated below the alignment with colored lines, as per the coloring of domains in Figure 2A and B. Boxes indicate residues lining the active sites.
(PDF)

Figure S4 **Consensus sequence alignment of plant RSHs, RelA and SpoT.** Domains are shown below the alignment with colored lines, as per the coloring of domains in Figure 2A and B. Where homology becomes undetectable, unalignable regions are indicated with backslashes and then not shown in subsequent lines.
(PDF)

Table S1 **Excel file showing all RSHs, the organisms that encode them, their ID numbers and database source.**
(XLSX)

Table S2 **Excel file showing the RSH complements of all sampled organisms, organized by taxonomy.**
(XLS)
