# Supplementary figures and images for: The RelA/SpoT Homolog (RSH) Superfamily: Distribution and Functional Evolution of ppGpp Synthetases and Hydrolases across the Tree of Life
Source: PLoS One. 2011 Aug 9;6(8):e23479. doi: 10.1371/journal.pone.0023479 (PMC3153485; doi:10.1371/journal.pone.0023479)

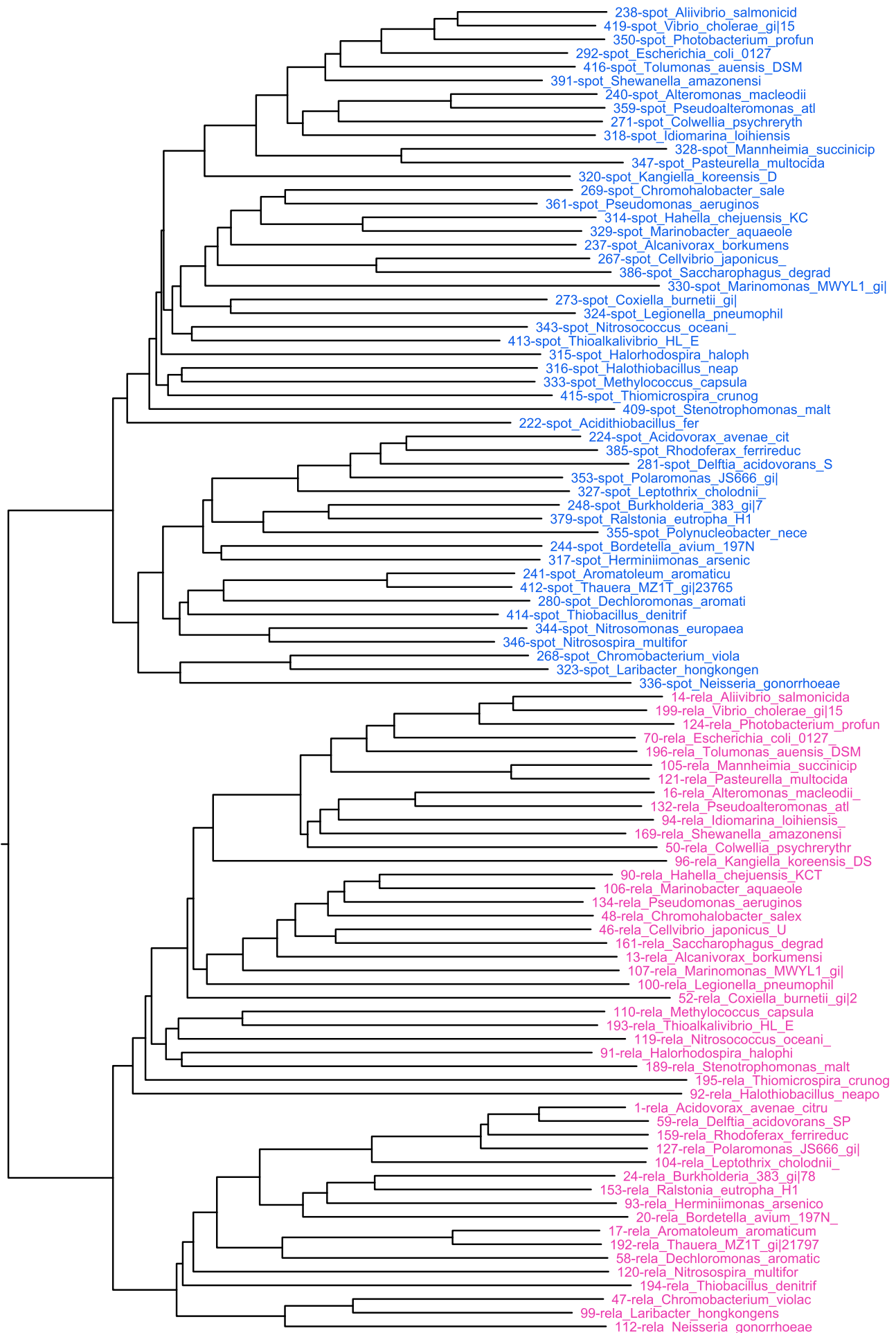

0.04

Supplement: Figure S1 — Phylogeny generated by DIVERGE for the analysis of site-specific rate shifts. RelA and SpoT are shown in pink and blue, respectively. (PDF) [file pone.0023479.s001.pdf]

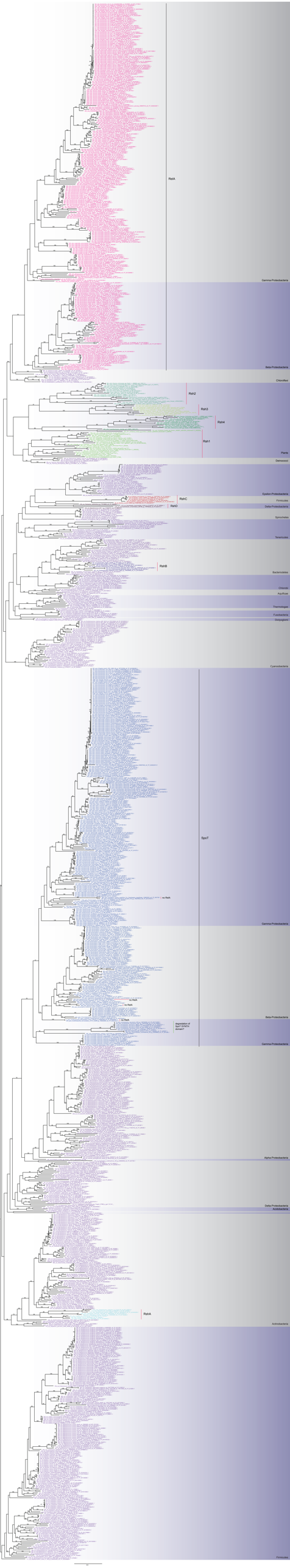

Supplement: Figure S2 — Maximum Likelihood phylogeny of the long RSHs using all alignable domains. The tree is generated from 699 amino acid positions. Bootstrap support values greater than 70% are shown on branches. All subgroups are labeled except for the paraphyletic Rel, which is shown in purple. Major taxonomic groups are indicated on the right. (PDF) [file pone.0023479.s002.pdf]
