# Supplementary material for: The RelA/SpoT Homolog (RSH) Superfamily: Distribution and Functional Evolution of ppGpp Synthetases and Hydrolases across the Tree of Life
Source: PLoS One. 2011 Aug 9;6(8):e23479. doi: 10.1371/journal.pone.0023479 (PMC3153485; doi:10.1371/journal.pone.0023479)
Supplement: Figure S3 — Sequence alignment of RelA[hS] and SpoT[HS]/[Hs] from Escherichia coli, Psychrobacter arcticum and Acinetobacter baumannii. Domains are indicated below the alignment with colored lines, as per the coloring of domains in Figure 2A and B. Boxes indicate residues lining the active sites. (PDF) [file pone.0023479.s003.pdf]

```
                                       10        20        30        40        50        60        70        80        90       100       110       120       130
RelA_Escherichia_coli          MVAVRSAHINKAGEF-------------------------------------------------------------------------------------------DPEKWIASLGIT-SQKSCECLAET
RelA_Psychrobacter_arcticum    MVKIREGLPLVDGQTTQADSATLAAQLVANQRSHQSANSYAQIPLDIKYQLATRKLHTTFDRSVNYAYYSHDSNLENDYLRDKILDESEAFSQQELEALDLDHINIDVPTWLDNVAKRIGQDSVPNLSAA
RelA_Acinetobacter_baumannii   MVTVREQLPEQLTELS-----------------------------------------------------------------------------EETTVEHAAETQVGLASWLDRVREILDGAELKQLEEV
SpoT_Escherichia_coli          MY--------------------------------------------------------------------------------------------------------LFESLNQLIQTYLPEDQIKRLRQA
SpoT_Psychrobacter_arcticum    MIQNLPKLSHPLVD--------------------------------------------------------------------------------------------DAQYNLLRSVGYLTGAERRDIVDA
SpoT_Acinetobacter_baumannii   MPGEEVSQ--------------------------------------------------------------------------------------------------AKQQLKLIIDPYLSVSEVEKVLAA

                                      140       150       160       170       180       190       200       210       220       230       240       250       260
RelA_Escherichia_coli          W--AYCLQQTQGHPDASLLLWRGVEMVEILSTLSMDIDTLRAALLFPLADANVVSEDVLRESVGKSVVNLIHGVRDMAAIRQLKAT------HTDSVSSEQVDNVRRMLLAMVDDFRCVVIKLAERIAHL
RelA_Psychrobacter_arcticum    CAFI-RKHMNTSASERSGAYVTGIGMTDILTYLYQDEDALVAAMLYRSARQSIISLNDIEKKFGADISTLVKDTLAMGQLSEIIESNKRLEDHFVNNQRDQLSNIYSMLISVTNDVRVVLIKLSERTFAM
RelA_Acinetobacter_baumannii   AHLTLQKELDSTVNHRSNTFATGVGMADILAHLHVDEDTLSAAMLYRSVREDITDIEEVKRKFGEQVYNLVKGTLAMGKLSELIEKNKRLEDHFNNNQREHLSGIYKMLISVTEDVRVVLIKLAERTYSL
SpoT_Escherichia_coli          YLVARDAHEGQTRSSGEPYITHPVAVACILAEMKLDYETLMAALLHDVIEDTPATYQDMEQLFGKSVAELVEGVSKLDKLK------------FRDKKEAQAENFRKMIMAMVQDIRVILIKLADRTHNM
SpoT_Psychrobacter_arcticum    CEFGDVAHIKDKRKSGEPYITHPIAVAEILAGFRLDRDTIIAAILHDTVEDTEVSDEQIETRYGKVVSRLVDGVTKLKSST-------------HNKQENKAATFHKILTATLADPRVLIIKLADRLHNM
SpoT_Acinetobacter_baumannii   CDFGDLAHTGITRKSGEPYILHPIAVSCILANMRLDPETLMAALLHDVIEDTQYTKDDIIERFGQTVAELVDGVTKLSQSS--------------DKEYNKAASFRKILQATLQDPRVIIIKLADRYHNM

                                      270       280       290       300       310       320       330       340       350       360       370       380       390
RelA_Escherichia_coli          REVKDAPEDERVLAAKECTNIYAPLANRLGIGQLKWELEDYCFRYLHPTEYKRIAKLLHERRLDREHYIEEFVGHLRAEMKAEGVKAEVYGRPKHIYSIWRKMQKKNLAFDELFDVRAVRIVAERLQDCY
RelA_Psychrobacter_arcticum    RELTFSNEDRQTRVAREVMTIYAPLAHRLGIAQLKWELEDLAFRYLAPDRYKEIAKLLSEKRSERESYIQRVQDRLNESLAESGIEGEVSGRVKHIYSIYRKMKLKGLSFDQLYDIRALRVLVTTPSDCY
RelA_Acinetobacter_baumannii   RELANSSRERQERVAREILTIYSPLAHRLGIAQLKWELEDLAFRYLAPDRYKEIASLLNEKRLEREHYIQFVIDRLKSELAAHGIEAEITGRAKHIYSIYRKMKSKNLSFDQLYDIRALRVLVNSVPECY
SpoT_Escherichia_coli          RTLGSLRPDKRRRIARETLEIYSPLAHRLGIHHIKTELEELGFEALYPNRYRVIKEVVKAARGNRKEMIQKILSEIEGRLQEAGIPCRVSGREKHLYSIYCKMVLKEQRFHSIMDIYAFRVIVNDSDTCY
SpoT_Psychrobacter_arcticum    STLDAVRPEKQRATAQETLDFYVPFARLMGLNDIADYIEVLCYRNLDSEMYNKISDKLLQHGLGRNFQREAIHRYLSIVLNNLDLDGMVKVLDNRV-VIFRQFFRNRGEINALLRHYAFEIVLDNIEACD
SpoT_Acinetobacter_baumannii   TTLGALRPDKRARIAQETFDIFVPMARLVGMNEMADNLENLCYQNLDLDMFDNVQNALLQTKPERCKYQSIWEQNLAELLHNYHIQGRIKKKNNNI-ELLRHFVKNEMDLQELTHSHAFEIVLQSIADCD

                                      400       410       420       430       440       450       460       470       480       490       500       510       520
RelA_Escherichia_coli          AALGIVHTHYRHLPDEFDDYVANPKPNGYQSIHTVVLGPGGKTVEIQIRTKQMHEDAELGVAAHWKYKEGAAAGGARSGHEDRIAWLRKLIA-WQE-------EMADSGEMLDEVRSQVFDD--------
RelA_Psychrobacter_arcticum    HVLGLVHGLWRYIPEQFDDYITNPKSNGYRSLHTAVI-AENKSLEVQIRTQEMHFEAELGMCAHVNYKEGL-KNKKDNYLNQRISSLRQLLS-INN-------QPRSSLSTGDELEETEFDDEEQLVDFD
RelA_Acinetobacter_baumannii   HSLGIVHQIWRHIPHQFDDYITNPKANGYRSLHTAVI-AENKSLEVQIRTHEMHDEAELGVCSHFNYKEGS--KNTDHSFNHRLHSLRAVLEHYQERNETTVHQNEDETEGFDQLQDFEGFE--------
SpoT_Escherichia_coli          RVLGQMHSLYKPRPGRVKDYIAIPKANGYQSLHTSMIGPHGVPVEVQIRTEDMDQMAEMGVAAHWAYKEHG--ETSTTAQIRAQRWMQSLLE-LQQ-------SAGSSFEFIESVKSDLFPD--------
SpoT_Psychrobacter_arcticum    KLAYYLIKKYQIADSHIADNIRRPLPGGNQSLTLIYE-RDNDFVKVTILTKQMQSAARLGVIGAEHASDVS-----QSVIQASLRNMKDLVD-EDCLSEGN-PDFATAVSTINELMDYLHSS--------
SpoT_Acinetobacter_baumannii   RLVAALKENFQVI--QYQDHIRRPLPGGNQSLMIKLK-GEKTTLSLTIQTELMRKAARFGVVLGENAPQTC-----RSAIQASMQNLNTLID-GEC-----------AKTTFNDLLDYLHQE--------

                                      530       540       550       560       570       580       590       600       610       620       630       640       650
RelA_Escherichia_coli          ---RVYVFTPKGDVVDLPAGSTPLDFAYHIHSDVGHRCIGAKIGGRIVPFTYQLQMGDQIEIITQKQPNPSRDWLNPNLGYVTTSRGRSKIHAWFRKQDRDKNILAGRQILDDELEHLGISLKEA----E
RelA_Psychrobacter_arcticum    ELERIYIFSRDGDITELPKGATVLDFAYYVHTQVGNRAQAARVNQRYVPLTYQLKTGEQVEIITKSSREPNRDWLVASLGYIHTNRARSKLRQWFNKQDRDKNIEIGRQMLSKELERLSVHPNSI---DL
RelA_Acinetobacter_baumannii   ---KIYVFSRDGDIKELPRGSTVLDFAYHVHTEVGNKCYAARVNQRYVPLTYTLKTGEQVEILTKKDREPNRDWLVNSLGYIKTARARDKLRHWFRQQDRSKNLEVGRELLNKELSRLAIHPKSI---DL
SpoT_Escherichia_coli          ---EIYVFTPEGRIVELPAGATPVDFAYAVHTDIGHACVGARVDRQPYPLSQPLTSGQTVEIITAPGARPNAAWL----NFVVSSKARAKIRQLLKNLKRDDSVSLGRRLLNHALGG-SRKLNEIPQENI
SpoT_Psychrobacter_arcticum    ---KIICYSPQGRAYELPQGATALDFAYAVGPMVGNVAVGANIDKKPAKLGTVVKNGQLVEIEVNSHSEPKAEWL----GFVVTNKARVEILRWFKDLSVADKQHHGRQALDRALKTYQKSLDDLTDSDW
SpoT_Acinetobacter_baumannii   ---KIWVYTPHGQLHELPQGATVVDFAYSASLFLGNHAVGAKVDGEIKPLSTPLVSGQVIEIITDVLATPNPDWL----SFINTQKARRALQHVLKDQDIEEQRLVGAQALSRALKLFNRSINDLSDADW

                                      660       670       680       690       700       710       720       730       740       750       760       770       780
RelA_Escherichia_coli          KHLLPRYNFNDVDELLAAIGGGDIRLNQMVNFLQSQFNKPSA--EEQDAAALKQLQQKSYTPQNRSKD--NGRVVVEGVGNLMHHIARCCQPIPGDEIVGFITQGRGISVHRADCEQLAELRSHAPERIV
RelA_Psychrobacter_arcticum    NDYTQHFNVNNTDDIVVGLVTGEIGLNQLTSHISRQLHLEPERSEEDF---------APTIDKRESGKLDAYKIQIDGLDNIEVGLAGCCHPVHGEPIAGYITLSRGVSVHNRGCPEYLRLIERDPEREI
RelA_Acinetobacter_baumannii   NDYSSHFNVKTGDDILVSLVSGDISLHALINQINRQMHLDQDEPELVL---------KPTLNPRASHTLSAHGILIDGLDNVELHIAQCCQPVHGESIAGYITLNRGVSIHKVLCSDYQRMIKQEPERAV
SpoT_Escherichia_coli          QRELDRMKLATLDDLLAEIGLGNAMSVVVAKNLQHG----------------------DASIPPATQS--HGHLPIKGADGVLITFAKCCRPIPGDPIIAHVSPGKGLVIHHESCRNIRGYQ-KEPEKFM
SpoT_Psychrobacter_arcticum    KNLTEWRGLTEKSALFEQISSGTLLPQLVVTRLFSDEVCDIQSQERIDDMTQPQQ-------------------LIVNASGVELDFANCCHPIYGDPIVGHLSR-HGLVVHRHKCFSLDDIRKDNPYQVI
SpoT_Acinetobacter_baumannii   LDLLQWRHIDNKDALFEQIAVGDLLPQLVANHLFANDKHPRAENSDR---------------------------LIQGTEGIDVKYAHCCNPILGDPIQGHLTR-RGLIVHRIRCHNLLHEQHLHPENIM

                                      790       800       810       820       830       840       850       860       870       880       890
RelA_Escherichia_coli          DAVWGESYS----------A--GYSLVVRVVANDRSGLLRDITTILANEKVNVLGVASRSDTKQQLATIDMTIEIYNLQVLGRVLGKLNQVPDVIDARRLHGS----------
RelA_Psychrobacter_arcticum    KAAWKIKSG----------R--YQPVDIHIEAYDRRGLLRDLTQIIDKENVNIRQVQTLSN-DDNIAFLKFHIEVSGLAHLSKLLAKLEQQHGILHARRAVA-----------
RelA_Acinetobacter_baumannii   EADWEMQPT----------R--GQSVQIVVEAYDRRGLLKDLTQVIFSDQINIRQVNTISE-ADGIANMKLLIEVKGLAQLSRLLARLEQQPGIISARRMIQGV---------
SpoT_Escherichia_coli          AVEWDKETA----------Q--EFITEIKVEMFNHQGALANLTAAINTTTSNIQSLNTEEK-DGRVYSAFIRLTARDRVHLANIMRKIRVMPDVIKVTRNRN-----------
SpoT_Psychrobacter_arcticum    QLRWHNDKAIKQSDSEDHGNKIRFPAYLKLSIAMSDEQISKVIYNLRQLNIGVEKVDVRGS------DTIIHIVVRSRNHLAQGIRELRSLLGFPNIMRLYQL----------
SpoT_Acinetobacter_baumannii   PLQWKADDV----------DDVRFTAYLAIYMAMNDEQVSDLIYQCRKNNAGVEMVHSNEQ------RTFVNIVVNNRKHIAKVIRDLRMHYGFPRIERLDAPAPQMEISKVS
```
