# Supplementary material for: The RelA/SpoT Homolog (RSH) Superfamily: Distribution and Functional Evolution of ppGpp Synthetases and Hydrolases across the Tree of Life
Source: PLoS One. 2011 Aug 9;6(8):e23479. doi: 10.1371/journal.pone.0023479 (PMC3153485; doi:10.1371/journal.pone.0023479)
Supplement: Figure S4 — Consensus sequence alignment of plant RSHs, RelA and SpoT. Domains are shown below the alignment with colored lines, as per the coloring of domains in Figure 2A and B. Where homology becomes undetectable, unalignable regions are indicated with backslashes and then not shown in subsequent lines. (PDF) [file pone.0023479.s004.pdf]

```
             10        20        30        40        50        60        70        80        90       100       110       120       130
        ....|....|....|....|....|....|....|....|....|....|....|....|....|....|....|....|....|....|....|....|....|....|....|....|....|....|
rsh4    ...lL...LKLalP.LQ..--....DGR..L.rAL.vA..LADL.MDA-----EVIsAGIL.e.me..-.vsl.evr..lG.....LLHe..RV...P.------rvd.-------LDDe.A..LR.F.L
rsh3    ....V.rA..fA...H---.GQ.R..Gd.Y..HCv.t..Il...l.....ra...V.A.lLHDVlDDT-......l...FG..v..lV..VSrl...NqllRR.RR..........L..Ee...LR.mlL
rsh2    .d..V..AF..A..AH---.G......d.yL.HCVETAv.LA.lG.ns-----..VaAGLLHDTlD.s-.m....i...F...V.DLV..VSklS.lSKL.R.nn.---------A....E..r...M.L
rsh1    E...V.eALkLAFeAH---dGQkRrSGEPfI.HPV.V.RIL.ELElD.-----ESi.AGLLHDTVEDTd.VTFE.lE..fG..VR.IVEGETKVSKl.........--------.s.qDvkadDL.QMFL
SpoT    lr.Ay..a..----AH---.GQ.R.SGEPYITHPvAVA.IlAem.LD.-----e.lmAALLHDViEDT-..T...l...FG..VAELVdGVSKLdKlkF..---------------k.EAQAENFRKMll
RelA    L............-.---...--............m..IL..L.mD.-----.tl.AAlLf.....-......l.e.fG..v..Lv.GV..m..i.........---------..-.Qve.lRkMLL

            140       150       160       170       180       190       200       210       220       230       240       250       260
        ....|....|....|....|....|....|....|....|....|....|....|....|....|....|....|....|....|....|....|....|....|....|....|....|....|....|
rsh4    -syyDiRAvvvELA.rLD.MRHlD.LPr.qQq..sLE.mqIYAPLAHAvG.G.LS.ELEDlsFr.LFP.SY..vd.WLrs.-------------------------------------------------
rsh3    .mV.dP.V.liKlADRLHNMRTiYAl...K...vA.ETL.VWCSLA.rLGmWavKsELEDLCFAVLeP..f..l.......W..................................................
rsh2    AM.-DARAVLIKLADRLHNMrTL.aLP..kQq.FA.ETleIfaPLANRLGI.s.K.qLEdL.FK.LnP.eh.eLS..L...----------------------------------------------------
rsh1    AMTEEVRvIIVKLADRLHNMRTL..MP..KQ..IA.ETL.VFAPLAkLLGmY.IKsELE.LSF.Y....dY..v.rRv..l---------------------------------------------------
SpoT    AM.rDiRVILiKLADR.HNMRTL.al..dKRRRIArETLEIYaPiAhRLGih.i..ELedL.F..lyP.Ry..l.k.Vk.A---------------------------------------------------
RelA    Amv.DvR.VlIkLA.Rl..LR.......e.r...ArE..dIYAPLANRLGI.QlKWELED.aFRyL.P..YK.IAk.L.Er--------------------------------------------------

            270       280       290       300       310       320       330       340       350       360       370       380       390
        ....|....|....|....|....|....|....|....|....|....|....|....|....|....|....|....|....|....|....|....|....|....|....|....|....|....|
rsh4    ----------------------....k.li..-------------..k.L..aLkA--------D..L..mV..v.V.GR.KSRyS.MKKLlrDGR.PEEVnDiLGLRVIL.Pr.G...------.e.G.
rsh3    ..........l.aV..FD.ls.R.........l..v......aL..L......L..El.i..Y...l----.--vtvS.RLKS.YS.y.KMKRK.v...ri.DARAlRvVVG-------D.........i
rsh2    ------------------------....mI.s-------------Ai.kLdk.L..---------..i----.-...L.GR.KSLYSIY.KM.KK...mDeIhDi..LRlIVe----------------.e
rsh1    ----------------------.K..E..l.e-------------A...L.e.iq.D.FL.--...v----.--..v....Ke.YSiyr...ks...i.ei..vAQlRIil....----..........
SpoT    ----------------------RGNRkEvi.k-------------I...i...L..---------..i----.--..v.GREK.LYSIY.KM..K...F..imDiY.FRviV.----------------.v
RelA    ----------------------RldRE.yI..-------------.v..L...L..---------..i----.--AeV.GRPKHIYSIWrKMq.K.L.F.eLyDvRAvRviv.----------------.l

            400       410       420       430       440       450       460       470       480       490       500       510       520
        ....|....|....|....|....|....|....|....|....|....|....|....|....|....|....|....|....|....|....|....|....|....|....|....|....|....|
rsh4    .ACYR..eIi..mWkevP.RTKDYIarPK.NGY.SLHmAv.vs..---...rPL----MElQIRT..M...A..G.ASHsLYK ///////////////////////////////////////////
rsh3    ..CY.LL.lvHrLw.PI.GEfDDYI.NPK.SGYqSLHTAV.---GP-DGa-------PlEVQiRT..MHe.AE.G.AA.WlYK.........---------------............S.S..---...
rsh2    .DCY.AL..VH.LW..VPGr.KDYI..PK.NGYqSLHTVV.---.e-..v-------PlEVQIRTkeMH..AEfG.AAHWRYKE......-------------------sfV.Q.VeWARWvlTWq.E..
rsh1    qiCYHVLGlVH.mW.PvP..mKDYIATPK.NGYqSLHT.Vi----P...e----.mF.LEv.IRTe.M..iAe.GIA...s...................R......n....a.Rv.WL.sIReWQeEFV
SpoT    ..CY..LG.lH.LYKP.PGrFKDYIAIPK.NGYQSLHTtL.---GP-.Gv-------PvEvQIRT.dM..mAe.GVAAHW.YK......------------------...Q.rA.-rWlqsLLelQ..a.
RelA    qDCY..LGiVH..w..iP.EFDDYiA.PK.NGYqSlHT.Vl---G.-.Gk-------.vEiQIRT..MH..AElGVAAHWkYKE.......----------------....Ye.kI.WLRqlL.W.eem.

            530       540       550       560       570       580       590       600       610       620       630       640       650
        ....|....|....|....|....|....|....|....|....|....|....|....|....|....|....|....|....|....|....|....|....|....|....|....|....|....|
rsh3    .........---------------------------..vql.--..--------------...v-..---------------------------G.....VVi-..d.---.G..mLV ///////////
rsh2    ---.k..............C.FP.H...C..s-y.-----.....d.PvfVIml..d.m.VqE.P..sT..DL.....--..............l..RlN...v......LkMGDVVEl ///////////
rsh1    ..mssR--------------------------EFV-DTiT.DLLG.-RVFVFTPkGE---i.NLPkGATVVDYAY.iH.eiGN.M.---------.AKVNG.lV-...HVL.NAEVVEI...-..l.s...
SpoT    ---.S.--------------------------EFi-E.VK.DLFPd-EiYVFTPkGr---Iv.LP.GAT.VDFAYAVHTDvGn.CV---------..rvn.....-PL...L.sG..VEIITa..A------
RelA    .---..--------------------------...-......v.dD-RvYV.TP.G.---VidLP.GaTPlDFAY.iHs.vGHRC.---------GAkv.GriV-P....Lq.G.qVEIiT.K..------

            660       670       680       690       700       710       720       730       740       750       760       770       780
        ....|....|....|....|....|....|....|....|....|....|....|....|....|....|....|....|....|....|....|....|....|....|....|....|....|....|
rsh1    ..R.kq...----.A.TRSAR.Ki.K.LrE...l.A..l..d.v...l.................................------------...-------------------
SpoT    -rPN.AWL.----FVvT.KAR..IR..LK..r..esv.LG.RLL..AL.......-----------.i........l.........dLl.dIGLG..ms.vvAr.L................-------
RelA    -.PSRDWLN..LGYl.t.RaRaKi..WF...drdkNi..Gr.lle.EL.r......----------------.......r......DdL...iG.Gdi.l..v...l......................

            790       800       810       820       830       840       850       860       870       880       890       900       910
        ....|....|....|....|....|....|....|....|....|....|....|....|....|....|....|....|....|....|....|....|....|....|....|....|....|....|
rsh1    -------------..............-------...............--..................................----v....i...DR.GllA.Vt..l...G..I...........-
SpoT    -------------l.I.G.eGm.v.fA.CC.PIPGD.Ii.hv..GkGlvvH...C..i-----......Pekwi-.v.W.......F..--.l.v.v.N.qG.LA.l...i.....sNI..i..e..--d.
RelA    ..............v.V.GV.nLm..lArCCqP.P.D.I.GfiTrGRGiSiHR.DC...----..L....PERvi-...W.......Y.v--.i.v.A.DR.GLLRDit.vla.EKvnVl.v.t.s..-..

            920       930       940       950       960
        ....|....|....|....|....|....|....|....|....|....|...
rsh1    .l..M.F.i....d-nL....s.v..i.GV...--S..C..............
SpoT    ....l...i.V.DR.HLA.vmR.lR.l..V.rv.R...---------------
RelA    ..A.m...iEv.....L.rvl..l.qv..Vi..rR.-----------------
```
